# A national survey of the clinical practice of surface-guided radiation therapy in Japan

**DOI:** 10.1093/jrr/rraf086

**Published:** 2026-01-14

**Authors:** Ryohei Yamauchi, Masahide Saito, Hironori Kojima, Yusuke Ueshima, Chie Kurokawa, Naoki Tohyama, Masahiko Kurooka, Shinobu Kumagai, Eriko Saito, Masataka Sakamoto, Takayuki Kanai, Hidekazu Suzuki, Tatsunori Saito, Tomoki Kitagawa, Makoto Sasaki, Hiroki Katayama, Yoshinobu Shimohigashi, Yoshifumi Oku, Naoki Hayashi, Takeshi Ohno, Hiroshi Onishi

**Affiliations:** Department of Radiation Oncology, St. Luke’s International Hospital, 9-1 Akashi-cho, Chuo-ku, Tokyo 104-8560, Japan; Department of Radiology, University of Yamanashi, 1110 Shimokato, Chuo City, Yamanashi 409-3898, Japan; Department of Radiology, Kanazawa University Hospital, 13-1 Takara-machi, Kanazawa City, Ishikawa 920-8641, Japan; Regional Creative Education Center, Hamamatsu University School of Medicine, 1-20-1 Handayama, Chuo-ku, Hamamatsu City, Shizuoka 431-3192, Japan; Faculty of Health Science, Juntendo University, 2-1-1 Hongo, Bunkyo-ku, Tokyo 113-8421, Japan; Department of Radiological Sciences, Komazawa University, 1-23-1 Komazawa, Setagaya-ku, Tokyo 154-8525, Japan; Department of Radiation Therapy, Tokyo Medical University Hospital, 6-7-1 Nishishinjuku, Shinjuku-ku, Tokyo 160-0023, Japan; Department of Radiology, Teikyo University Hospital, 2-11-1 Kaga, Itabashi-ku, Tokyo 173-8606, Japan; Department of Radiation Oncology, Chiba Tokushukai Hospital, 2-11-1 Takanedai, Funabashi City, Chiba 274-8503, Japan; Department of Radiology, Hamamatsu University School of Medicine, 1-20-1 Handayama, Chuo-ku, Hamamatsu City, Shizuoka 431-3192, Japan; Department of Radiation Oncology, Tokyo Women’s Medical University, 8-1 Kawada-cho, Shinjuku-ku, Tokyo 162-8666, Japan; Department of Radiological Technology, Faculty of Medical Technology, Teikyo University, 2-11-1 Kaga, Itabashi-ku, Tokyo 173-8605, Japan; Department of Radiology, Seirei Hamamatsu General Hospital, 2-12-12 Sumiyoshi, Chuo-ku, Hamamatsu City, Shizuoka 430-8558, Japan; Department of Radiation Oncology, Aichi Cancer Center, 1-1 Kanokoden, Chikusa-ku, Nagoya City, Aichi 464-8681, Japan; Division of Clinical Radiology Service, Kyoto University Hospital, 54 Shogoin-Kawahara-cho, Sakyo-ku, Kyoto 606-8507, Japan; Division of Clinical Radiology, Department of Medical Technology, Kagawa University Hospital, 1750-1 Ikenobe, Miki-cho, Kita-gun, Kagawa 761-0793, Japan; Department of Radiological Technology, Kumamoto University Hospital, 1-1-1 Honjo, Chuo-ku, Kumamoto City, Kumamoto 860-8556, Japan; Department of Radiology, Kagoshima University Hospital, 8-35-1 Sakuragaoka, Kagoshima City, Kagoshima 890-8520, Japan; Division of Medical Physics, School of Medical Sciences, Fujita Health University, 1-98 Dengakugakubo, Kutsukake-cho, Toyoake City, Aichi 470-1192, Japan; Faculty of Life Sciences, Kumamoto University, 4-24-1 Kuhonji, Chuo-ku, Kumamoto City, Kumamoto 862-0976, Japan; Department of Radiology, University of Yamanashi, 1110 Shimokato, Chuo City, Yamanashi 409-3898, Japan

**Keywords:** surface-guided radiation therapy, survey, clinical practices

## Abstract

The purpose of this study was to evaluate the diffusion of surface-guided radiation therapy (SGRT), implementation of quality control and quality assurance strategies, established clinical workflows and user perceptions regarding the benefits and limitations of SGRT in routine practice. From October to December 2024, we surveyed 880 radiotherapy institutions in Japan regarding institutional characteristics, quality assurance/quality control, computed tomography simulation, treatment procedures and general questions regarding SGRT. The survey was distributed via mailing list and through vendors, and administered via Google Forms. A total of 292 institutions responded, corresponding to a response rate of 33%. Ninety-eight institutions reported introducing SGRT, and 50 institutions had introduced it after 2022. The highest usage rate of SGRT in breast treatment was 87%. Approximately half of the institutions performed daily checks of SGRT and radiation isocenter coincidence, as well as static accuracy, whereas 6% did not perform these checks at all. The primary functions of the SGRT system were patient positioning (94%), respiratory management (78%), patient monitoring (76%) and skin marker–less techniques (69%). Many institutions reduced or eliminated skin marking, citing simplified workflows and reduced setup time. Many respondents observed that SGRT implementation reduced both setup and treatment times for breast/chest, abdomen/pelvis and extremity procedures. SGRT has been widely embraced in Japan, offering notable clinical and workflow benefits. However, because participation in this survey was voluntary, the results may overrepresent institutions with greater awareness or adoption of SGRT. Greater standardization, broader insurance coverage and ongoing technological advancements are essential to fully realize its advantages.

## INTRODUCTION

Surface-guided radiation therapy (SGRT) constitutes a sophisticated form of image-guided radiation therapy (IGRT) that integrates recent advances in surface imaging technology to refine the precision of radiation delivery [1–3]. A principal benefit of SGRT is its non-ionizing nature, which removes the need for supplemental radiation exposure during treatment. Consequently, SGRT is routinely used with linear accelerators across a range of clinical applications, including patient setup, intra-fraction motion monitoring, deep inspiration breath hold protocols and automated beam gating in response to patient displacement. SGRT’s adaptability to diverse anatomical treatment sites further bolsters its clinical utility.

Recently, professional organizations, such as the American Association of Physicists in Medicine (AAPM) and the European Society for Radiotherapy and Oncology (ESTRO), have released comprehensive guidelines, notably the AAPM TG-302 report [4] and the ESTRO-Advisory Committee for Radiation Oncology Practice (ACROP) guideline [5]. These publications offer an in-depth discussion of SGRT, encompassing clinical and technical workflows, risk evaluation for potential failures, commissioning and routine quality assurance (QA) and quality control (QC). Using these guidelines, healthcare institutions can streamline the implementation and optimization of SGRT within their practices.

SGRT is widely adopted worldwide in radiation therapy, but the bulk of existing data on its clinical use is limited to studies performed by AAPM [6] and ESTRO [7] prior to the publication of current guidelines. Recent reports include a study by Seravalli *et al.*, which focused on pediatric patients [8]. Therefore, no studies on the clinical use of SGRT have been performed since the publication of these guidelines, and the clinical adoption status of emerging SGRT systems, recent technological developments and SGRT platforms specific to certain regions remains unclear. In particular, when SGRT systems are developed and distributed primarily within a specific country or region (e.g. VOXELAN in Japan), internationally established guidelines may not be directly applicable without contextual adaptation. This is not because the fundamental principles of SGRT differ but because variations in registration algorithms, reference surface options and linac/couch integration interfaces can influence system accuracy and the implementation of treatment and QA/QC procedures.

This study aimed to bridge this knowledge gap—specifically, the lack of updated information regarding current clinical practices, system adoption and local adaptation of SGRT—by investigating its application in Japanese radiotherapy centers. In particular, we aimed to examine the diffusion of SGRT technology, implementation of QA/QC strategies, established clinical workflows and user perceptions regarding the benefits and limitations of SGRT in routine practice.

## MATERIALS AND METHODS

### Survey design and analysis procedures

From October to December 2024, we performed a comprehensive survey to evaluate the current application of SGRT in Japan. Invitations to complete the survey were disseminated to all radiotherapy institutions (*n* = 880) via the official mailing lists of the Japanese Society for Radiation Oncology, Japanese Society of Medical Physics, Japanese College of Medical Physics and Japan Society of Radiological Technology, as well as several regional radiotherapy-related organizations. We also collaborated with vendors distributing SGRT systems in Japan, including EURO MEDITECH Co., Elekta AB, Brainlab, Varian Medical Systems and ERD Co., who shared the survey invitation with their affiliated institutions. The survey encompassed all medical institutions offering radiotherapy services, regardless of whether they had implemented an SGRT system.

A standardized invitation was used across all channels. The message described the objective of the survey, ensured that responses would remain anonymous and voluntary and included a link to the online Google Forms questionnaire. The questionnaire comprised both multiple-choice and open-ended items, with an estimated completion time ranging from 5 to 30 minutes, depending on the respondent’s background and experience. Each institution was queried only once, and no patient-identifiable data were collected. Participation was voluntary and restricted to institutions that consented to join the study. All procedures were approved by the Institutional Review Board of our institution (approval number 24-RC041, approval date 12 June 2024).

The questionnaire was designed to investigate the clinical use patterns of SGRT and was divided into six main sections: (i) institutional characteristics (e.g. annual patient volume, number of treatment machines and number of SGRT systems); (ii) QA/QC protocols; (iii) computed tomography (CT) simulation; (iv) treatment planning; (v) treatment procedures (e.g. treatment sites where SGRT is used, primary purpose of SGRT, reference surface imaging, gating thresholds); and (vi) general questions (e.g. plans for future SGRT adoption and barriers to its implementation). The survey items were formulated on the basis of prior publications and consultations with radiation oncologist (RO), medical physicists (MPs) and radiation therapists (RTTs) experienced with various SGRT platforms. Subsequent to these consultations, modifications and additions were made, and a pilot test was performed to confirm the face validity of the survey.

To ensure data integrity, submissions were reviewed to eliminate duplicate, inconsistent or contradictory responses within the same institution. Where multiple entries arose from the same individual at a given clinic, only the most recent submission was retained. If a single institution submitted multiple entries from different personnel, priority was granted on the basis of the respondent’s professional role (RO, MP, RTT, in that order). Data processing and cross-checking were performed using Excel (Microsoft Corporation, Redmond, WA, USA) by teams comprising at least two authors.

## RESULTS

### Survey response rate and prevalence of SGRT systems

Table 1 presents an overview of the participating institutions, including their use of SGRT and general information regarding SGRT implementation. A total of 292 institutions responded, corresponding to a response rate of 33.2%. The majority of respondents were RTTs (73.3%, *n* = 214), followed by MPs (23.3%, *n* = 68) and ROs (3.4%, *n* = 10).

**Table 1:** Summary of respondent characteristics and general surface-guided radiotherapy system information about prevalence and clinical implementation

	*n*	%
**Respondent (*n* = 292)**		
Radiation oncologist	10	3.4
Medical physicist	68	23.3
Radiation therapist	214	73.3
**SGRT system in treatment room (*n* = 292)**		
No	194	66.4
Yes (one system)	83	28.4
Yes (two systems)	15	5.1
**SGRT system in CT room (*n* = 98)**		
No	83	66.2
Yes (one system)	15	28.7
**Number of patients treated per year (*n* = 292)**		
<99 (with SGRT)	20 (5)	6.8 (5.1)
100–199 (with SGRT)	56 (15)	19.2 (15.3)
200–299 (with SGRT)	60 (18)	20.5 (18.4)
300–399 (with SGRT)	40 (11)	13.7 (11.2)
400–499 (with SGRT)	30 (10)	10.3 (10.2)
500–749 (with SGRT)	42 (22)	14.4 (22.4)
>750 (with SGRT)	44 (17)	15.1 (17.3)
**Year of installation (*n* = 98)**		
Prior to 2015	3	3.1
2015–18	2	2.0
2018–19	11	11.2
2019–20	12	12.2
2020–21	8	8.2
2021–22	12	12.2
2022–23	14	14.3
2023–24	16	16.3
2024-present	20	20.4
**Clinical implementation after acceptance test (*n* = 98)**		
<1 month	38	38.8
1–6 months	34	34.7
7–12 months	8	8.2
13–24 months	3	3.1
>24 months	3	3.1
Not used clinically	8	8.2
Don’t know	4	4.1
**What took the longest to start using the SGRT system in clinical practice (*n* = 90)**		
Commissioning (mechanical)	15	16.7
Commissioning (clinical)	10	11.1
Determination of clinical workflow	60	66.7
Consensus building	3	3.3
Other	2	2.2
**SGRT system in treatment room (*n* = 98)**		
AlignRT (VisionRT)	28	28.6
Catalyst (C-RAD)	37	37.8
ExacTrac Dynamic (BrainLab)	10	10.2
IDENTIFY (Varian)	11	11.2
VOXELAN (ERD)	11	11.2
Multiple vendor	1	1.0

*(continued)*

Among the 292 institutions that responded, 28.4% (*n* = 83) used one SGRT system, while 5.1% (*n* = 15) used two. Of these, only 15.3% had the SGRT system installed in CT rooms. In response to a query on SGRT usage duration (*n* = 98), 20.4% reported usage for less than 1 year, 42.8% for 1–3 years, and 36.7% for >3 years. After conducting acceptance tests, 38.8% of the respondents commenced clinical usage of SGRT within 1 month, and 34.7% did so within 6 months. The most frequently cited factor delaying SGRT implementation was ‘determination of clinical workflow’. Conversely, 8.2% (*n* = 8) indicated having access to SGRT systems but refrained from clinical use.

The majority of institutions used conventional C-arm linear accelerators: 50.4% (*n* = 57) from Varian Medical Systems (Palo Alto, CA, USA) and 40.7% (*n* = 46) from Elekta AB (Stockholm, Sweden). The vendors of the SGRT systems were AlignRT (VisionRT, London, UK), 28.6%; Catalyst (C-RAD, Uppsala, Sweden), 37.8%; ExacTrac Dynamic (Brainlab, Munich, Germany), 10.2%; IDENTIFY (Varian Medical Systems, Palo Alto, CA, USA), 11.2%; and VOXELAN (ERD Co., Okayama, Japan), 11.2%. Supplementary Fig. 1 shows the year of first SGRT system implementation by the vendor.

### Acceptance, commissioning and QA/QC

Table 2 provides a summary of the QA/QC-related questions and responses. Commissioning was performed by MPs in 36.8% of institutions and/or by certified RTTs in 66.3%. For acceptance test, commissioning and QA/QC, vendor-provided guidelines were used most frequently (58.2%), followed by AAPM TG-302 (33.7%), AAPM TG-147 (25.5%) and ESTRO-ACROP (20.4%). Institutions that installed SGRT systems after 2022 were more likely to reference AAPM TG-302, while references to ESTRO-ACROP were observed in a smaller but steady proportion of institutions. References to vendor guidelines remained consistently high across all years (Supplementary Fig. 2). As shown in Supplementary Fig. 3, institutions with the highest annual patient volumes (>750 cases per year) tended to reference SGRT-specific guidelines more frequently than others. In contrast, no clear differences were observed among other institutions, where vendor-provided guidelines remained the most commonly referenced materials. Vendor guidelines were the most frequently cited references across all SGRT systems, while the use of AAPM and ESTRO-ACROP guidelines varied significantly by vendor (Supplementary Fig. 4).

**Table 1:** Continued.

	*n*	%
**Location in clinic (*n* = 113)**		
Varian C-arm	57	50.4
Varian Halcyon	4	3.5
Elekta C-arm	46	40.7
Tomotherapy	3	2.7
CyberKnife	0	0.0
Particle therapy	0	0.0
Other	3	2.7
**SGRT system in CT room (*n* = 15)**		
SimRT (VisionRT)	2	13.3
Sentinel (C-RAD)	11	73.3
IDENTIFY (Varian)	0	0.0
VOXELAN (ERD)	2	13.3
**Establishing an alternative treatment workflow in the case of system failure (*n* = 98)**		
Yes	64	65.3
No	34	34.7

**Table 2:** Summary of answers to questions regarding QA/QC protocols

	*n*	%
**Staff for commissioning (*n* = 98)**		
Radiation oncologist	0	0.0
Medical physicist	53	36.8
Certified radiation therapist	65	66.3
Non-certified radiation therapist	26	18.1
**References used for acceptance, commissioning and QA/QC (*n* = 98)** ^**a**^		
AAPM TG-302	33	33.7
AAPM TG-147	25	25.5
AAPM TG-76	4	4.1
AAPM TG-142	18	18.4
ESTRO-ACROP	20	20.4
Vendor’s guideline	57	58.2
Publication	20	20.4
Nothing	6	6.1
Don’t know	4	4.1
Other	9	9.2
**Warm-up time to ensure stability of the SGRT system (*n* = 85)**		
None	41	48.2
10 minutes	11	12.9
20 minutes	5	5.9
30 minutes	16	18.8
40 minutes	1	1.2
50 minutes	2	2.4
60 minutes	7	8.2
Don’t know	2	2.4
**QA protocol in clinic (*n* = 98)**		
Yes	32	32.7
No	33	33.7
In progress	33	33.7

^a^Multiple answers allowed.

**Fig. 1:**
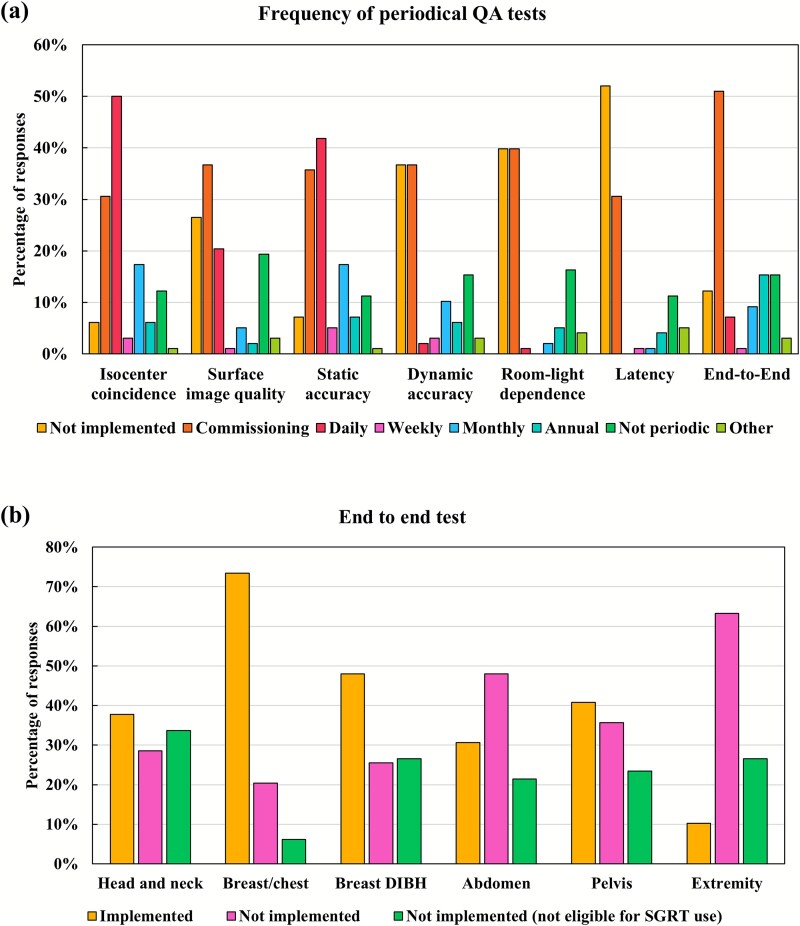
Distributions of (**a**) frequency of QA tests and (**b**) end-to-end tests per treatment site.

Approximately half of the institutions performed daily checks of SGRT isocenter and radiation isocenter coincidence, as well as static accuracy, whereas 6% (*n* = 6) did not perform these checks at all (Fig. 1a). These six institutions used SGRT for patient positioning but had not implemented this or any other periodic QA procedure. Two facilities had only recently installed their SGRT systems; one was unsure how to perform the checks, and one reported that QA was deemed unnecessary. More than 40% of the institutions did not evaluate dynamic accuracy, room-light impact or latency time. Among the 51 institutions that did not evaluate latency, 16 facilities (31.3%) reported using SGRT for patient monitoring involving gating. End-to-end testing was performed at 89% of the institutions (Fig. 1a), with a particularly high proportion (72%) simulating breast or chest treatments (Fig. 1b).

**Fig. 2:**
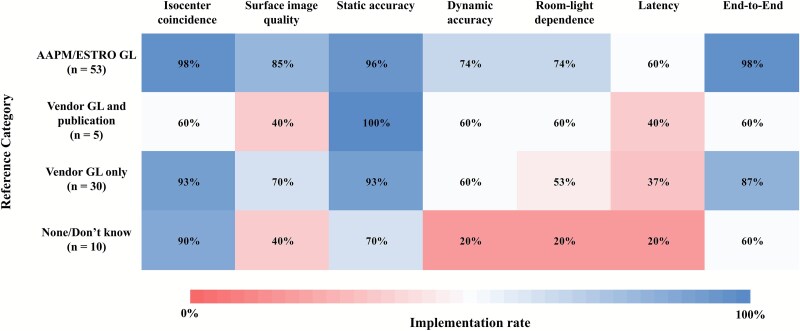
Heatmap of SGRT QA implementation rates stratified by reference category. Institutions were grouped based on the materials used to guide QA procedures: international guidelines (AAPM and/or ESTRO-ACROP), vendor guidelines plus scientific publications, vendor guidelines only or no reference. GL, guideline.

To better understand these variations, QA implementation rates were further analyzed by the type of guidance followed, as shown in Fig. 2. Institutions that referred to AAPM and/or ESTRO-ACROP guidelines demonstrated consistently higher implementation rates across all QA items. In contrast, institutions that did not refer to international guidelines but relied on vendor guidelines, instead, exhibited moderate implementation rates overall, with relatively high adherence for isocenter coincidence and static accuracy tests but lower implementation for dynamic accuracy, room-light dependence and latency assessments. The lowest implementation rates were observed in institutions that did not refer to any guidelines or were unsure of the references used.

Supplementary Fig. 5 shows the distributions of QA test implementation by vendor. While tests of isocenter coincidence and static accuracy were routinely performed across most SGRT systems, Catalyst and ExacTrac Dynamic users reported the highest rates of daily implementation. In contrast, advanced QA items, such as dynamic accuracy, room-light dependence, latency and end-to-end tests were often performed only during commissioning or were not implemented in some systems. AlignRT users demonstrated relatively higher implementation rates for these advanced QA items compared with users of other systems.

**Fig. 3:**
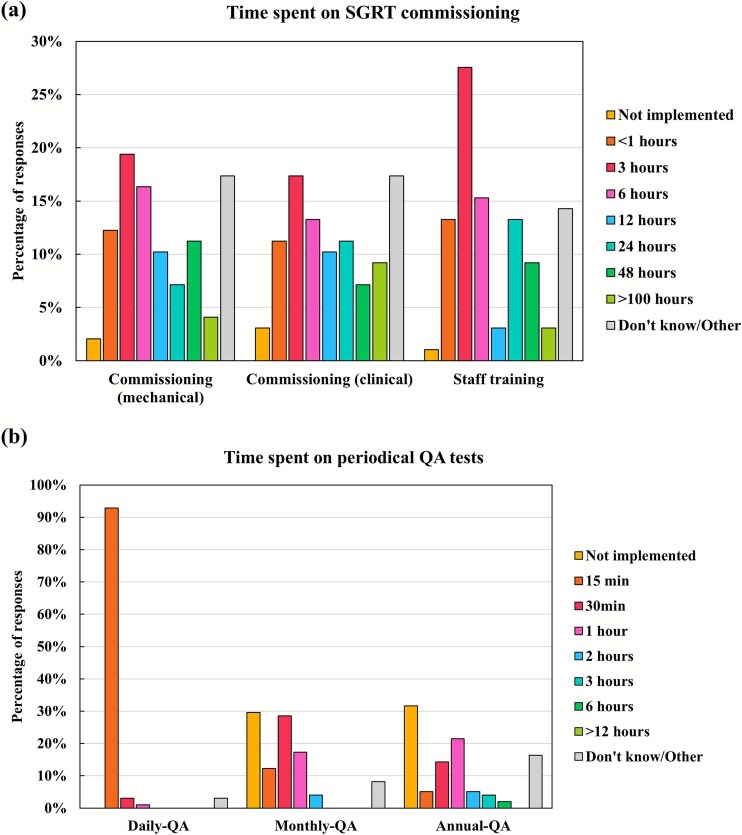
Distributions of (**a**) time spent on commissioning and (**b**) time spent on periodic QA tests.

Regarding the time required for SGRT commissioning—including mechanical commissioning, clinical commissioning and staff training—the most frequently reported total was 3 hours (Fig. 3a). While time allocations varied widely across SGRT vendors, AlignRT, Catalyst and VOXELAN users were most likely to report longer durations (>24 hours) for mechanical and clinical commissioning. Conversely, no differences among the systems were observed in completing these activities in under 6 hours (Supplementary Fig. 6).

For periodic QA procedures, the most common durations were 15 minutes on a daily basis, 30 minutes monthly and 1 hour annually (Fig. 3b). Monthly and annual QA durations varied considerably among vendors. Notably, nearly all ExacTrac Dynamic users spent >30 minutes on monthly QA, whereas 20%–40% of the users of other systems did not perform monthly QA (Supplementary Fig. 7).

Supplementary Fig. 8 summarizes the warm-up time required to ensure system stability. While some vendors (e.g. IDENTIFY, VOXELAN) frequently reported long warm-up times, ~60% of other institutions did not implement a warm-up period.

Overall, only 34% of institutions implemented a QA protocol, while 34% were in the process of creating one. Differences between systems were small, but among IDENTIFY users, fewer institutions had not created a QA protocol (Supplementary Fig. 9).

### SGRT system use in the CT room

Among the 15 institutions with SGRT systems installed in CT simulation rooms (Table 1), 13 had implemented SGRT clinically. In 12 of these 13 institutions, clinical use began within 6 months after acceptance testing. The most frequently cited challenges during implementation were determination of the clinical workflow (*n* = 8), mechanical commissioning (*n* = 4), and clinical commissioning (*n* = 3). During CT simulation, eight institutions reported using the SGRT system to acquire surface data, with seven of these using the Sentinel system (C-RAD, Uppsala, Sweden). Additionally, 13 institutions reported using the CT-room SGRT system for respiratory motion management.

### CT simulation and treatment planning

Table 3 presents a summary of the relevant questions and responses regarding CT simulation and treatment planning for institutions equipped with SGRT. Among all SGRT-equipped institutions, 10.2% adjusted CT imaging parameters, such as scan speed or tube voltage, during CT simulation. Additionally, 36.7% reported modifying immobilization devices or patient positioning; 24.5% eliminated skin markings and 29.6% partially reduced skin marking. When stratified by whether SGRT was installed in the CT room, these values showed minor differences. Among SGRT-equipped institutions with a CT-room SGRT in clinical use (*n* = 13), 7.7% adjusted imaging parameters and 53.8% modified immobilization or positioning; 24.1% eliminated markings, and 27.7% partially reduced marking. Among those without a CT-room SGRT or not using it clinically (*n* = 85), the corresponding values were 10.6%, 36.5%, 23.5% and 27.1%, respectively. The presence of SGRT in the CT room may slightly influence decisions related to patient setup and marking, although overall differences were modest.

**Table 3:** Summary of answers to questions about CT simulation and treatment planning

	*n*	%
**Change in CT simulation protocol (*n* = 98)**		
No	88	89.8
Yes (scan speed)	3	3.1
Yes (scan range)	4	4.1
Yes (scan settings)	1	1.0
Yes (other)	2	2.0
**Change in fixtures and/or treatment positions during CT simulation (*n* = 98)** ^**a**^		
No	62	63.3
Yes (change arm up devices)	8	8.2
Yes (use vacuum cushions)	11	11.2
Yes (no longer use vacuum cushions)	0	0
Yes (no longer use body shells)	9	9.2
Yes (change to open mask; all patients with head cases)	4	4.1
Yes (change to open mask (some patients with head cases)	14	14.3
Yes (change to open mask (all patients with head and neck cases)	2	2.0
Yes (change to open mask (some patients with head and neck cases)	7	7.1
Yes (no longer use body shells; all patients)	0	0.0
Yes (no longer use body shells; some patients)	13	13.3
Yes (change patient positions)	5	5.1
**Skin marking during CT simulation (*n* = 98)**		
Change to skin marker-less	24	24.5
Reduce skin marker	29	29.6
No change as before (with skin marking)	43	43.9
No change as before (without skin marking)	2	2.0
**Change in body contour (e.g. default settings of delineation HU) in TPS (*n* = 98)** ^**a**^		
Yes	7	6.9
No	53	35.6
No (but confirmed)	36	52.5
No (because body contour information is not used)	5	5.0
**Consideration of adequacy of margins (*n* = 98)**		
Yes (larger)	0	0.0
Yes (smaller)	3	3.1
Yes (but no change)	41	41.8
No	54	55.1

^a^Multiple answers allowed.

### Clinical use of SGRT

#### Common treatment sites

Among the 98 responding institutions, breast radiotherapy represented the most prevalent SGRT application (86.7%, *n* = 85), followed by abdomen and pelvis (56.1%, *n* = 55), extremities (44%, *n* = 43), head and neck (39.8%, *n* = 39) and head stereotactic radiosurgery (28.6%, *n* = 28).

#### Main SGRT applications

**Fig. 4:**
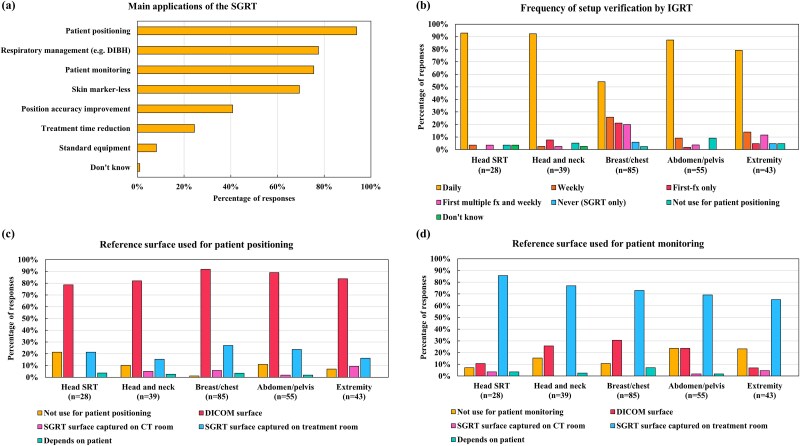
Distributions of (**a**) the main application of an SGRT system, (**b**) different setup verification protocols, (**c**) reference surface used for patient positioning and (**d**) reference surface used for intra-fraction monitoring per treatment sites. SGRT, surface-guided radiation therapy; IGRT, image-guided radiation therapy; SRT, stereotactic radiotherapy; DICOM, digital imaging and communications in medicine; CT, computed tomography; DIBH, deep inspiration breath hold.

The primary functions of the SGRT system were patient positioning (93.9%), respiratory management (e.g. deep inspiration breath hold) (77.6%), patient monitoring (75.5%) and skin marker-less techniques (69.4%) (Fig. 4a).

#### Complementary IGRT

Institutions using SGRT were additionally queried about their use of IGRT to confirm SGRT setup positions. Daily IGRT in conjunction with SGRT was the most commonly reported protocol across the majority of the treatment centers (Fig. 4b). For the breast/chest region, daily IGRT was occasionally omitted for position verification.

#### Reference surface

Most respondents reported that the digital imaging and communication in medicine (DICOM) surface generated from the treatment planning CT (hereafter referred to as the DICOM surface) was used as the primary reference surface for daily patient setup across all treatment sites. By contrast, an SGRT camera–captured surface acquired at the first fraction (the SGRT surface) was rarely used for patient positioning. However, the SGRT surface served as the principal reference for patient monitoring during treatment, regardless of the treatment site (Fig. 4c and d). Supplementary Fig. 10 further illustrates vendor-specific patterns. Since no marked site-specific differences were observed, data from all treatment sites were averaged to provide overall vendor-wise tendencies. While reference surface selection for patient positioning was consistent across systems—with the DICOM surface being the default—monitoring practices showed greater variability. AlignRT users more frequently used the DICOM surface even for monitoring, whereas VOXELAN users often reported not using any reference surface for this purpose.

### Skin marking

**Fig. 5:**
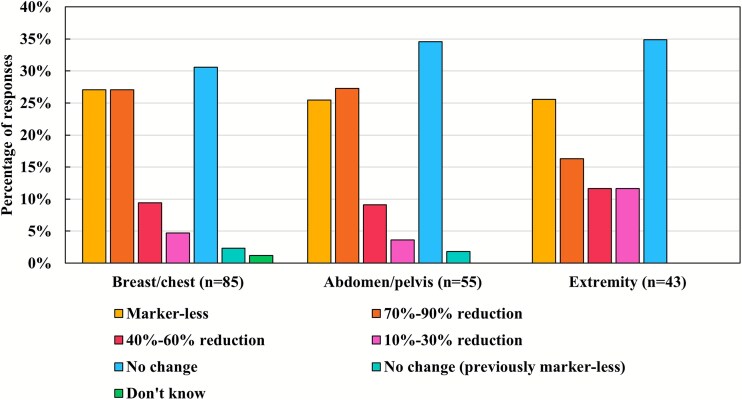
Changes in skin marking by treatment site after the introduction of an SGRT system. SGRT, surface-guided radiation therapy.

With the implementation of SGRT, ~25% of the institutions eliminated tattoos or skin marks entirely across all anatomical regions (Fig. 5). Approximately 40% reported a partial reduction. When prompted for further reasons why skin marking was not completely discarded, many respondents (*n* = 26/53) indicated that marking remains integral for patient positioning, visual confirmation of the irradiation field and electron beam alignment, with SGRT serving as an auxiliary method. Additional justifications included the use of skin marking as a contingency in cases of SGRT system malfunction (*n* = 7), lack of operational experience (*n* = 4) and future plans to diminish reliance on skin marking (*n* = 6). Some respondents also highlighted system-related constraints, including limited SGRT field of view (*n* = 4), and expressed reservations about SGRT setup accuracy (*n* = 8). Supplementary Fig. 11 presents vendor-specific distributions of changes in skin marking practices following SGRT implementation. Institutions using AlignRT, Catalyst and ExacTrac Dynamic were more likely to report reductions in skin marking, with AlignRT users in particular showing the highest adoption rate of marker-less workflows. In contrast, IDENTIFY and VOXELAN users reported no change in marking practices more frequently than users of other systems, regardless of the treatment site.

### Workflow and treatment time changes

**Fig. 6:**
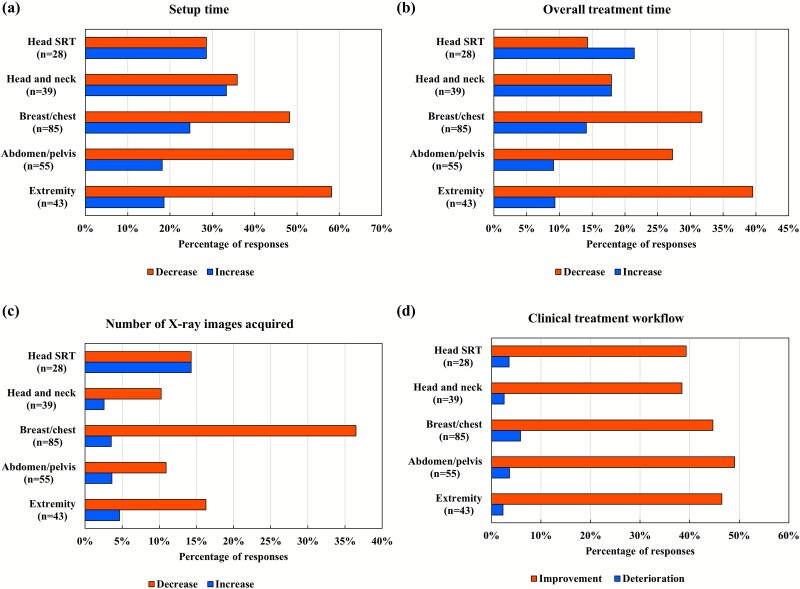
Changes in (**a**) setup time, (**b**) overall treatment time, (**c**) number of X-ray images acquired and (**d**) clinical workflow by treatment site after introducing an SGRT system. SGRT, surface-guided radiation therapy; SRT, stereotactic radiotherapy.

Regarding workflow and treatment durations, many respondents indicated that SGRT implementation reduced both setup and treatment times for breast/chest, abdomen/pelvis and extremity procedures (Fig. 6). By contrast, for head stereotactic radiotherapy and head and neck therapies, the proportion of institutions reporting an increase in setup or treatment times was comparable to or greater than those reporting a decrease. Notably, 36.5% of the institutions experienced a reduction in the number of IGRT sessions for breast/chest treatments. Across all treatment sites, ~40% of the institutions indicated overall improvement in the treatment workflow.

### Perception of advantages and limitations of SGRT

Among the survey participants, 33.0% (*n* = 96) reported that they are considering or planning to acquire an SGRT system in the future. In response to the question, ‘Do you believe SGRT should immediately become a standard for patient positioning and position matching, with all linear accelerators equipped with SGRT technology?’, 63.3% answered ‘yes’ or ‘maybe’. Additionally, 92.5% (*n* = 270) identified cost as a barrier to SGRT adoption. Many respondents urged societies and manufacturers to ‘establish national guidelines’, ‘translate international guidelines into Japanese’, ‘hold training courses’ and ‘increase insurance coverage’. The questions and responses concerning the perception of SGRT’s advantages and limitations are summarized in Table 4.

**Table 4:** Summary of responses to questions regarding the advantages and limitations of SGRT systems

	*n*	%
**Do you consider introducing the SGRT system in the future? (*n* = 291)**		
Installed	102	35.1
Under consideration (newly)	80	27.5
Under consideration (addition)	16	5.5
No	88	30.2
Other	5	1.7
**Do you consider that the SGRT system will be standard care and that all linacs should be equipped with SGRT? (*n* = 292)**		
Yes	97	33.2
No	69	23.6
Maybe	88	30.1
Don’t know	38	13.0
**Agreement on necessity for national SGRT guidelines (*n* = 292)**		
Agree	174	59.6
Disagree	3	1.0
Maybe	81	27.7
Don’t know	34	11.6
**Activities that manufacturers and academic societies should carry out regarding the SGRT system (*n* = 292)** ^**a**^		
Japanese translation of SGRT guidelines	164	56.2
Establishment of national guideline	204	69.9
Seminars	206	70.5
Practical training courses	153	52.4
Briefings on the differences between manufacturers	98	33.6
On-site support	52	17.8
Expansion of medical fee coverage	199	68.2
Other	5	1.7

^a^Multiple answers allowed.

## DISCUSSION

This survey examined the prevalence of SGRT systems in Japan, focusing on differing approaches to commissioning, QA/QC and clinical application. Our findings provide a snapshot of current practices and highlight perceived advantages and limitations of SGRT from the viewpoints of both experienced and prospective users.

Although the overall response rate was 33.2%, data from the Japanese Society for Radiation Oncology structural survey (as of December 2023) indicated that 137 institutions were equipped with SGRT systems [9], and our follow-up with vendors (as of September 2024) identified 170 SGRT-equipped institutions. On the basis of these findings, the survey responses covered ~72% and 58% of SGRT-equipped institutions, respectively. Therefore, the findings related to SGRT-specific clinical practices are likely to be reasonably representative. Nonetheless, this study has limitations regarding generalizability. Because participation was voluntary, institutions that had implemented or were actively considering SGRT may have been more motivated to respond. Consequently, the dataset may overrepresent facilities with higher awareness or usage of SGRT. This potential response bias should be considered when interpreting our findings, particularly for survey items that address general institutional practices beyond SGRT implementation. Nevertheless, this study offers valuable insights into SGRT’s implementation in Japan in 2024, highlighting existing challenges and potential areas for further development.

The release of two major international guidelines—AAPM TG-302 and ESTRO-ACROP— has helped standardize the adoption and implementation of SGRT in clinical practice [4, 5]. In Japan, SGRT systems have recently proliferated rapidly, and half of the surveyed institutions introduced SGRT following publication of these guidelines. As shown in Supplementary Fig. 2, recent adopters (post-2022) referred more frequently to international guidelines, whereas earlier adopters relied predominantly on vendor-provided materials. Among the 57 institutions citing vendor guidelines, 30 (52.6%) used them exclusively, while the remainder supplemented these guidelines with AAPM or ESTRO-ACROP documents. Although vendor guidelines are often used for QA/QC, they rarely contain detailed procedures, and many institutions likely relied on training documents, handouts or supplementary checklists instead. Despite the availability of international guidelines, engagement with these documents—particularly for commissioning and QA—remained limited. Language barriers and the lack of nationally standardized resources were frequently cited as obstacles, underscoring the need for translated and localized guidance. Importantly, QA implementation rates varied significantly depending on the type of reference materials consulted. As shown in Fig. 2, institutions that referred to formal guidelines demonstrated substantially higher adherence across all QA domains. In contrast, institutions that relied solely on vendor documents or lacked any reference materials showed markedly lower implementation rates. These results highlight the importance of accessible, standardized national guidelines to support consistent QA practices across institutions.

A further concern in the survey results was the wide variation in protocols—both in scope and frequency—for SGRT commissioning and QA/QC among institutions. This variation was not related to the period that an SGRT system had been in service. Consequently, commissioning time varied considerably, although most centers spent <6 hours on mechanical tasks; significantly less than the ≥25 hours reported by the ESTRO survey for approximately half of their surveyed sites. Implementation rates for isocenter consistency and static accuracy were high (94% and 93%, respectively), mirroring findings from the ESTRO survey [7]. By contrast, rates for more complex assessments—dynamic accuracy, room-light impact and latency—were lower, with 40% of the institutions omitting these tests, compared with 72% in the ESTRO survey. Furthermore, among institutions that did not evaluate latency, nearly one-third reported using SGRT for gated irradiation. This suggests a potential mismatch between clinical application and QA validation. Additionally, some respondents expressed uncertainty about how to perform specific QA procedures or considered them unnecessary. While not explicitly stated, technical or institutional constraints, such as lack of tools, unclear protocols or insufficient vendor support, may also contribute to underutilization in some settings. These findings highlight the need for structured, vendor-independent procedural manuals that go beyond listing QA items and include step-by-step instructions, acceptance criteria and workflow integration strategies. Appendix III of ESTRO-ACROP offers a useful foundation, but further elaboration and regional adaptation are warranted to support consistent SGRT implementation [5].

The end-to-end testing implementation rate was 89%, exceeding the rates of 74% reported in the AAPM survey [6] and 80% in the ESTRO survey [7]. This high uptake reflects the strong emphasis placed on end-to-end testing in international SGRT guidelines [4, 5]. Most institutions performed end-to-end tests for breast cancer, likely because SGRT is highly suited to breast radiotherapy and widely adopted in clinical practice. In Japan, reimbursement policies that allow billing for SGRT only in breast irradiation may further drive this trend. Moreover, numerous publications provide practical guidance for breast applications, accelerating SGRT uptake [1–3]; consequently, SGRT use for breast cases exceeds 90%. Owing to the close alignment between breast radiotherapy workflows and SGRT, daily X-ray imaging for positioning is often unnecessary, and many institutions have adopted workflows that omit IGRT verification.

Regarding reference surface selection (DICOM vs. SGRT-captured), a clear dichotomy emerged between patient positioning and patient monitoring. Many institutions used the DICOM surface to align patients with their CT simulation position, while the SGRT surface was used mainly to track patient motion from the position verified by IGRT. These findings are consistent with those in previous surveys [7]. Differences in reference surface use for patient monitoring also varied by vendor. AlignRT users showed a higher tendency to use DICOM surfaces for monitoring compared with users of other systems, suggesting an emphasis on consistency with the planning CT and confidence in the system’s positional accuracy. Conversely, VOXELAN users reported lower use of monitoring compared with users of other systems, which may be because of limited integration with linac systems.

The vendor-specific trends may reflect not only differences in system specifications but also historical factors and implementation maturity. AlignRT and Catalyst have been available since the early stages of SGRT adoption, and their users may have benefited from the broader dissemination of QA/QC protocols. This accumulated knowledge could explain the wider adoption of comprehensive QA procedures, longer QA durations and more confident transitions to marker-less workflows in institutions using these systems compared with institutions using other systems. In contrast, newer or region-specific systems, such as ExacTrac Dynamic and VOXELAN, may face limitations because of shorter adoption periods or less established support infrastructures, possibly contributing to lower QA implementation rates compared with users of other systems. To support more consistent and evidence-based SGRT implementation, broader dissemination of international guidelines and development of national consensus-based protocols are warranted. These efforts would also help improve the quality and safety of SGRT workflows across diverse clinical environments.

Notably, commissioning duties were frequently undertaken by MPs and certified RTTs, which contrasts with European practice, in which RTTs are typically not involved in technical commissioning, a task handled mainly by MPs [7]. However, in Japan, the definition and employment of ‘medical physicist’ is not standardized. Certification as a qualified MP does not necessarily indicate full-time clinical physicist status or formal employment. Similarly, some RTTs also hold medical physics certification, and in practice, responsibilities for commissioning and QA may be shared or reassigned on the basis of institutional needs. While this differs from European norms, such involvement may be clinically advantageous, as RTTs are well positioned to integrate SGRT procedures into daily patient care workflows.

Twenty-five percent of centers in Japan have transitioned to a fully marker-free workflow, closely matching the 23% reported in prior investigations [7]. Moreover, roughly 40% have adopted partial marking reduction—double the 20% cited in earlier reports [7] and likely reflecting the expanding literature documenting the efficacy of SGRT-guided setups. Nevertheless, skin-mark-based positioning remains prevalent in Japan, driven by limited SGRT experience, device constraints, contingency planning for system failures and lingering doubts about SGRT’s spatial accuracy. Given that tattoos and skin markers can induce psychological distress [10, 11], the marker-free approach enabled by SGRT offers a compelling means to mitigate these concerns, and many adopters are actively refining clinical workflows to realize this benefit.

An expanding body of literature underscores SGRT’s utility, prompting 33% of institutions in this survey to consider adopting the technology. Moreover, 63% of survey respondents believe that SGRT has the potential to become a new standard of care, aligning with recent publications on the future trajectory of SGRT. The chief concerns regarding SGRT use are up-front and ongoing expenses. Under the current Japanese social medical insurance, SGRT-related billing is largely restricted to breast indications, compelling some institutions to cover the costs associated with other indications themselves.

This survey revealed that SGRT is widely implemented and successful in various clinical settings, but several avenues remain for further improvement. First, cost-effectiveness analyses in diverse healthcare systems are critical because they may optimize resource allocation and expand insurance coverage for SGRT beyond breast cases. Second, technical standardization, which encompasses commissioning protocols, QA/QC procedures and vendor-specific guidelines, may enhance interinstitutional consistency and reduce inconsistencies in clinical practice. Finally, continued advances in surface imaging technology may further refine the role of SGRT in radiation therapy. Collaborative efforts among clinicians, researchers, professional societies and manufacturers are essential to address these challenges and fully realize the potential of SGRT to improve patient care and outcomes.

This survey is the first, to our knowledge, to demonstrate that SGRT system implementation has enhanced treatment times and streamlined clinical workflows, particularly in breast, abdomen/pelvis and extremity treatments. Streamlined workflows encompassing shortened setup times, reduced radiation exposure, intra-fraction motion monitoring and fewer skin markings have benefited both clinical teams and patients. Conversely, a subset of institutions, particularly those performing head stereotactic radiotherapy and head and neck treatments, reported increases in setup and overall treatment times. These increases may be explained by several factors. Traditionally, these regions rely on rigid immobilization devices, such as thermoplastic masks, enabling efficient setup. The addition of SGRT surface alignment introduces a new step that may increase preparation time. Moreover, SGRT’s capacity for continuous monitoring may lead to the detection of intra-fraction motion or positioning discrepancies that were previously overlooked, prompting additional image verification or corrective action. While such processes may extend treatment duration, they likely contribute to improved accuracy and clinical safety.

In conclusion, this survey demonstrated that SGRT is widely used in Japan for patient positioning and monitoring, offering clinical advantages, such as reduced skin marking and shorter setup and treatment times. However, QA/QC protocols vary between institutions, and the use of international guidelines remains limited. Accordingly, there is a strong demand for domestic guidelines and Japanese translations of international guidelines. High costs and limited insurance coverage are major barriers to broader SGRT adoption. To maximize SGRT’s benefits, further standardization, wider reimbursement, continued technological innovation and collaboration among stakeholders are essential.

## Supplementary Material

Supplementary_file_20251031_rraf086
