# Supplementary material for: A national survey of the clinical practice of surface-guided radiation therapy in Japan
Source: J Radiat Res. 2026 Jan 14;67(1):134–47. doi: 10.1093/jrr/rraf086 (PMC12856029; doi:10.1093/jrr/rraf086)
Supplement: Supplementary_file_20251031_rraf086 [file supplementary_file_20251031_rraf086.docx]

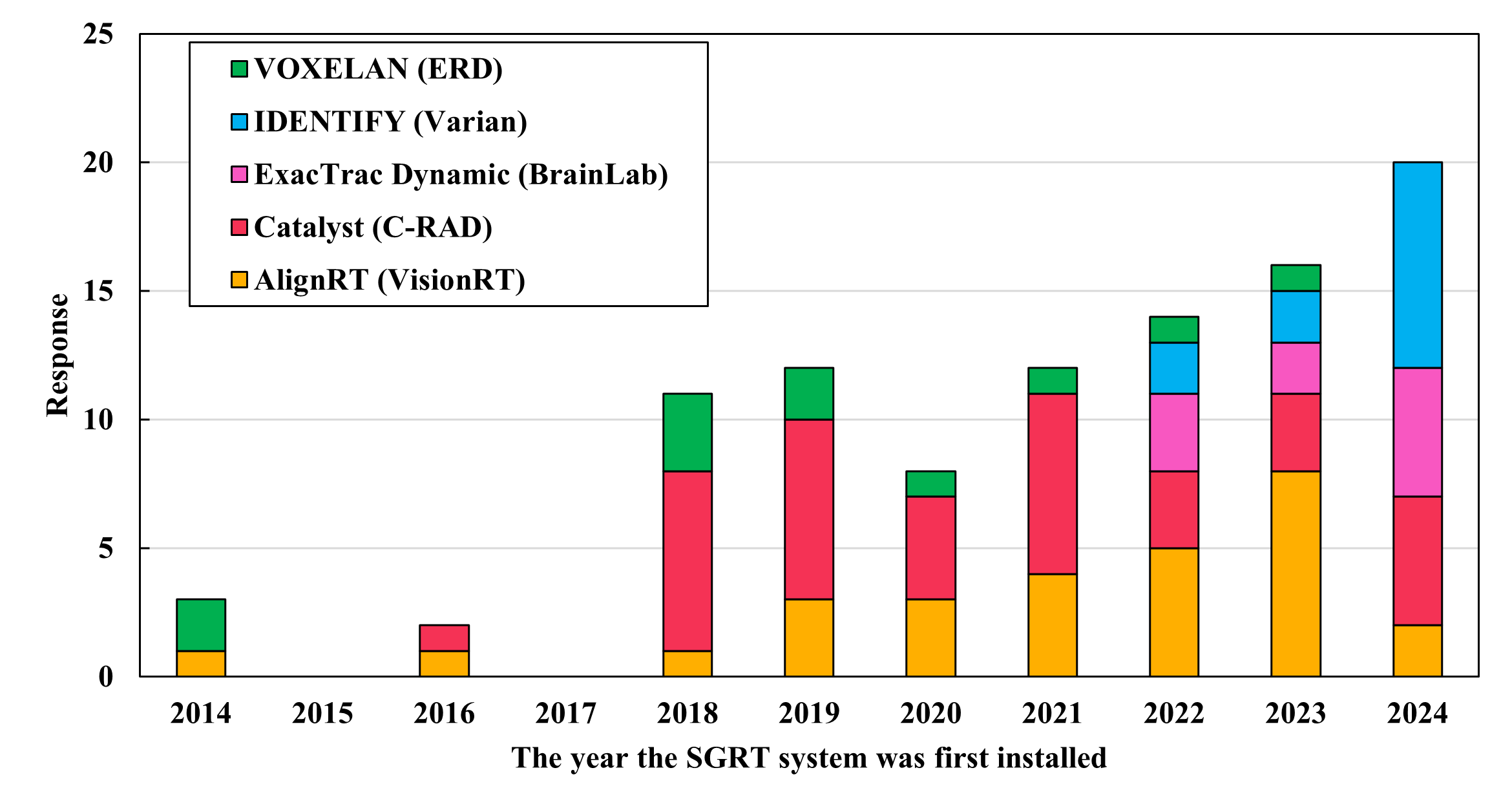


Supplementary figure 1 Year of first SGRT system installation in treatment room, by vendor.


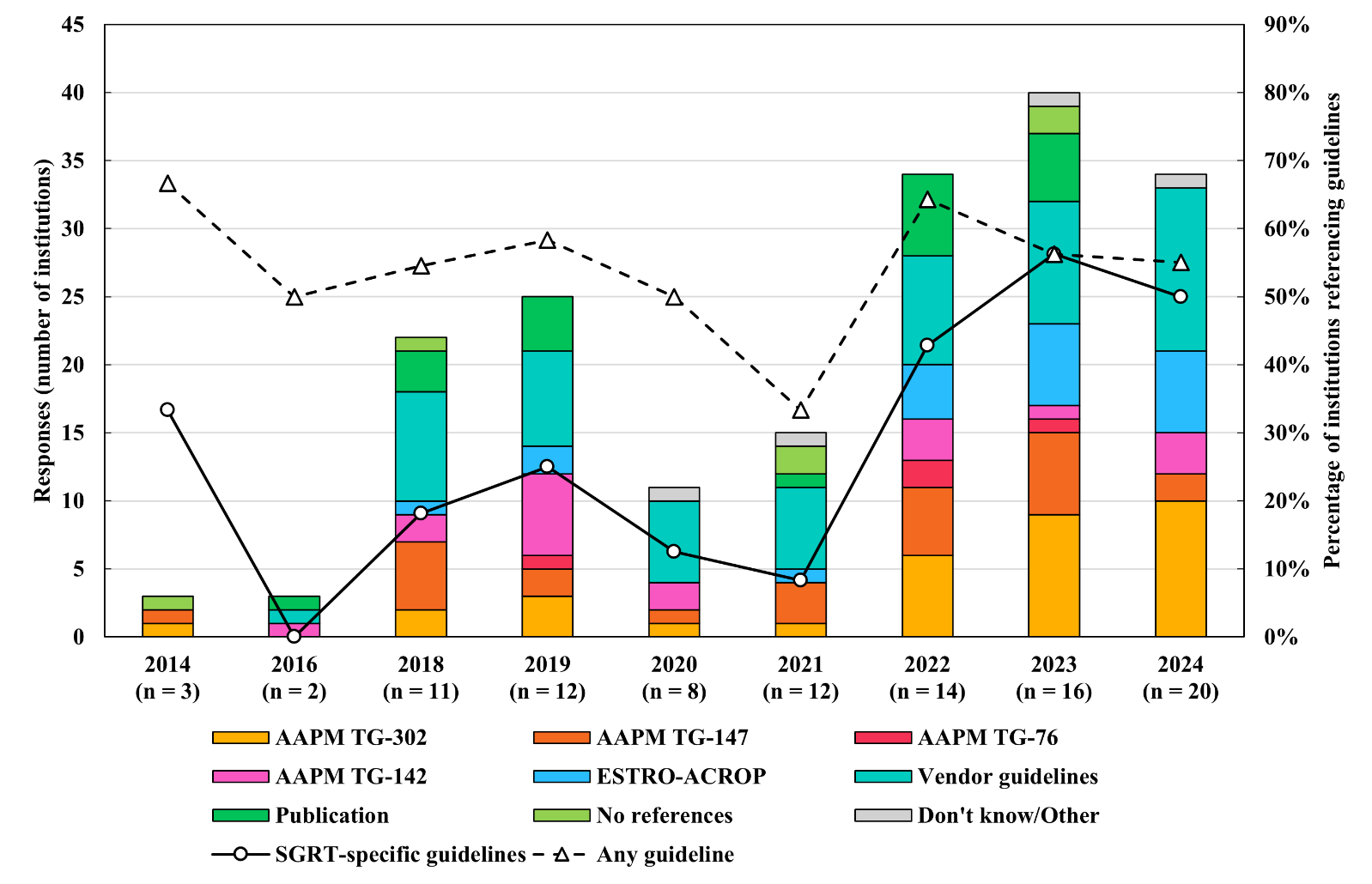


Supplementary figure 2 References used for acceptance test, commissioning, and QA/QC, grouped by year of SGRT system installation.

Stacked bars indicate the number of institutions referencing each guideline or document type.
The solid line (○) represents the proportion of institutions referencing SGRT-specific guidelines (AAPM TG-302 or ESTRO-ACROP), and the dashed line (△) indicates the proportion referencing any guideline (AAPM TG-302, TG147, TG-76, TG-142, or ESTRO-ACROP).


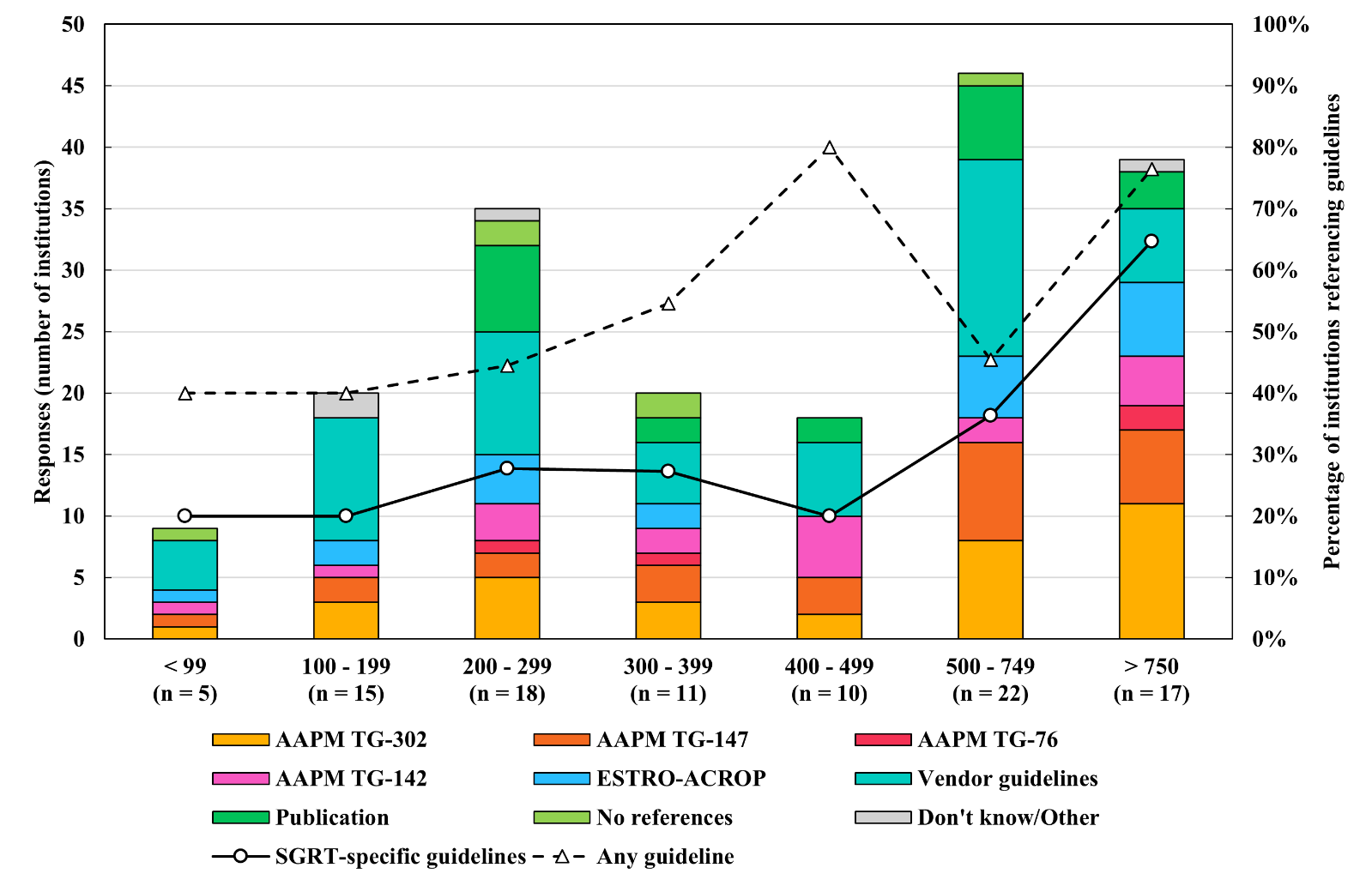


Supplementary figure 3 References used for acceptance testing, commissioning, and QA/QC procedures, stratified by the number of patients treated per year.
Stacked bars indicate the number of institutions referencing each guideline or document type.
The solid line (○) represents the proportion of institutions referencing SGRT-specific guidelines (AAPM TG-302 or ESTRO-ACROP), and the dashed line (△) indicates the proportion referencing any guideline (AAPM TG-302, TG147, TG-76, TG-142, or ESTRO-ACROP).


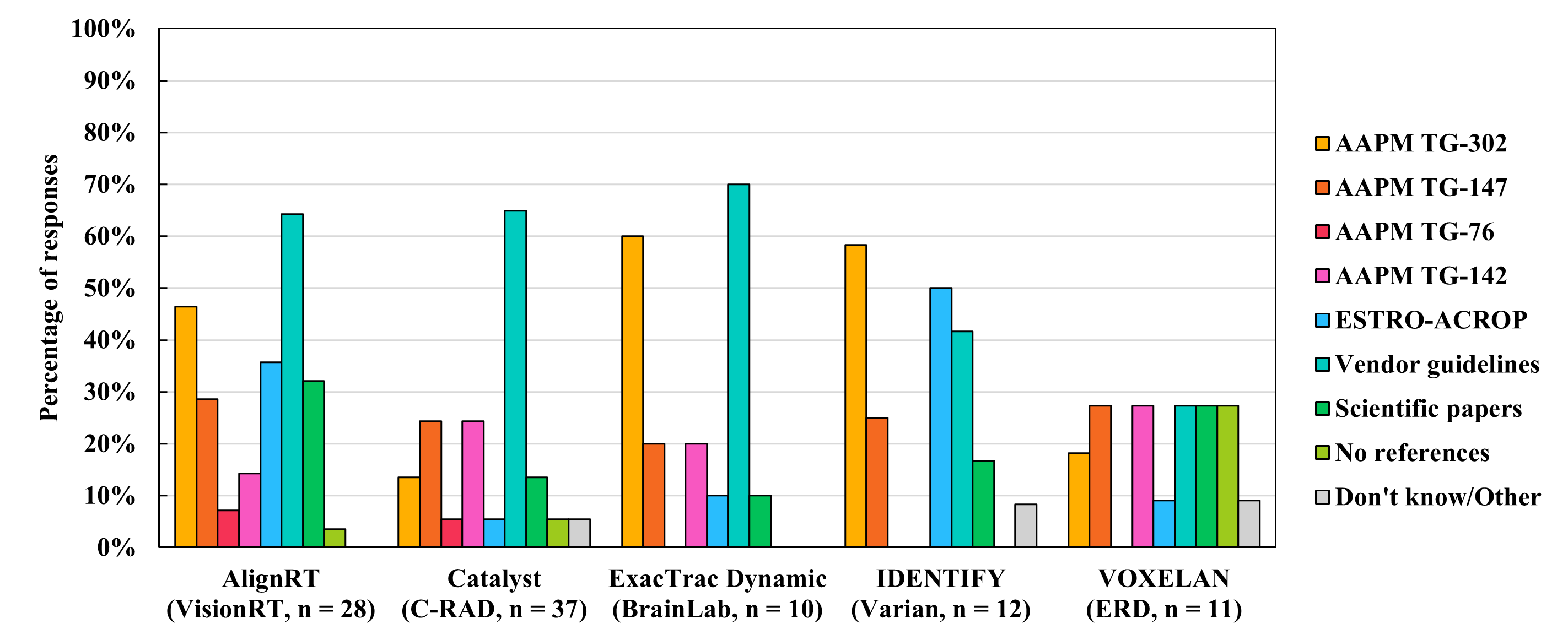


Supplementary figure 4 References used for acceptance test, commissioning, and QA/QC, by vendor.

1. Isocenter coincidence


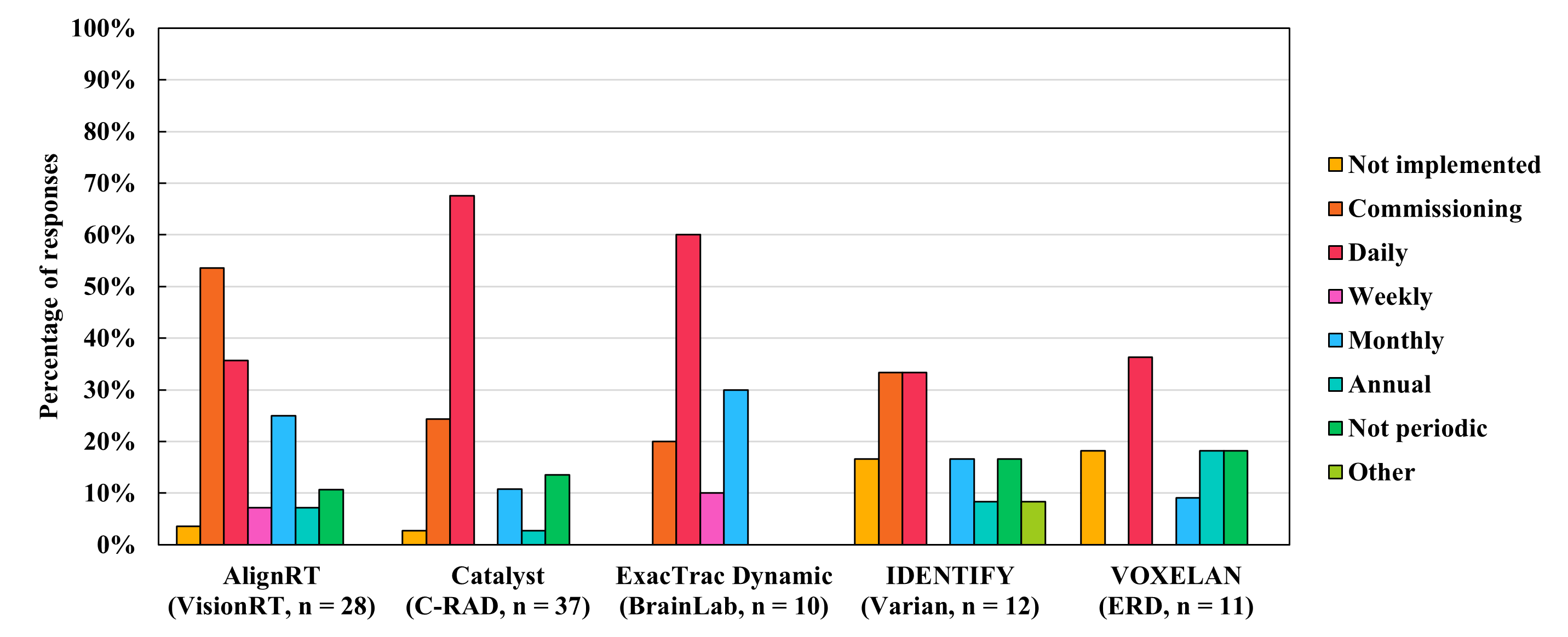


1. Surface image quality


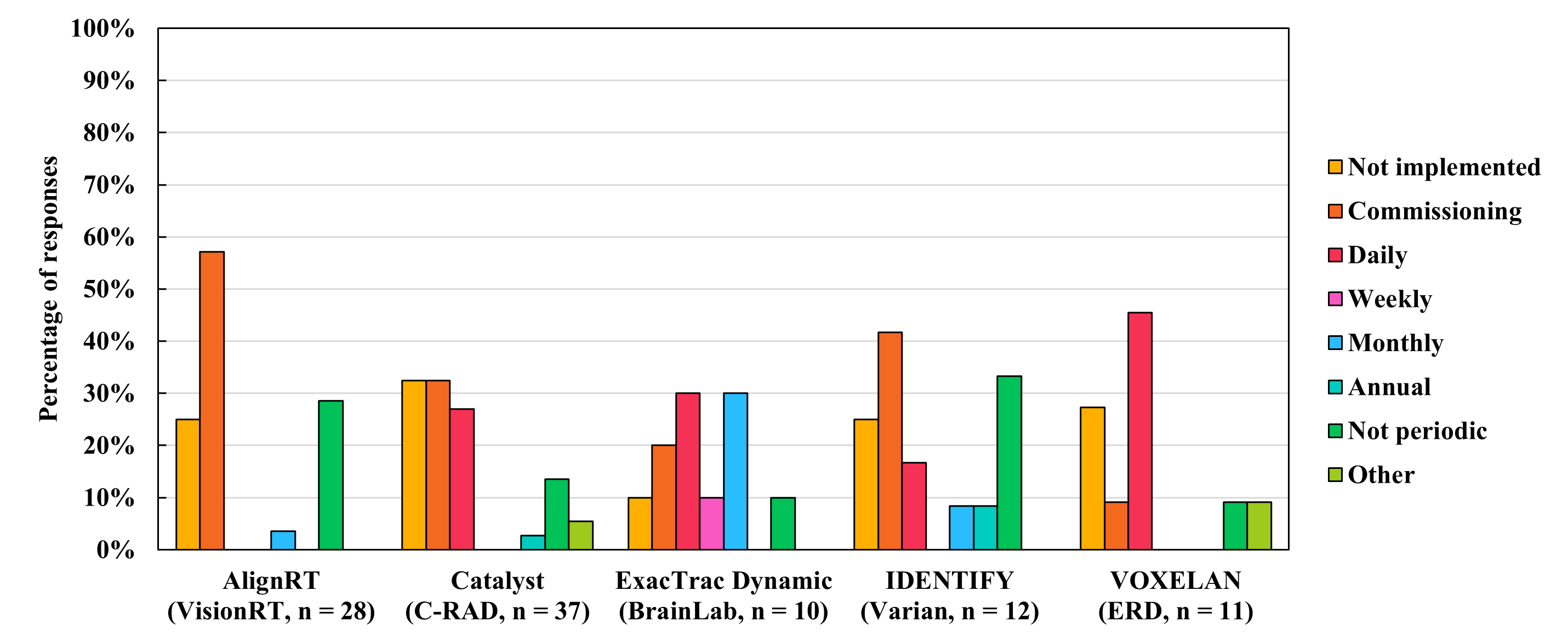


1. Static accuracy


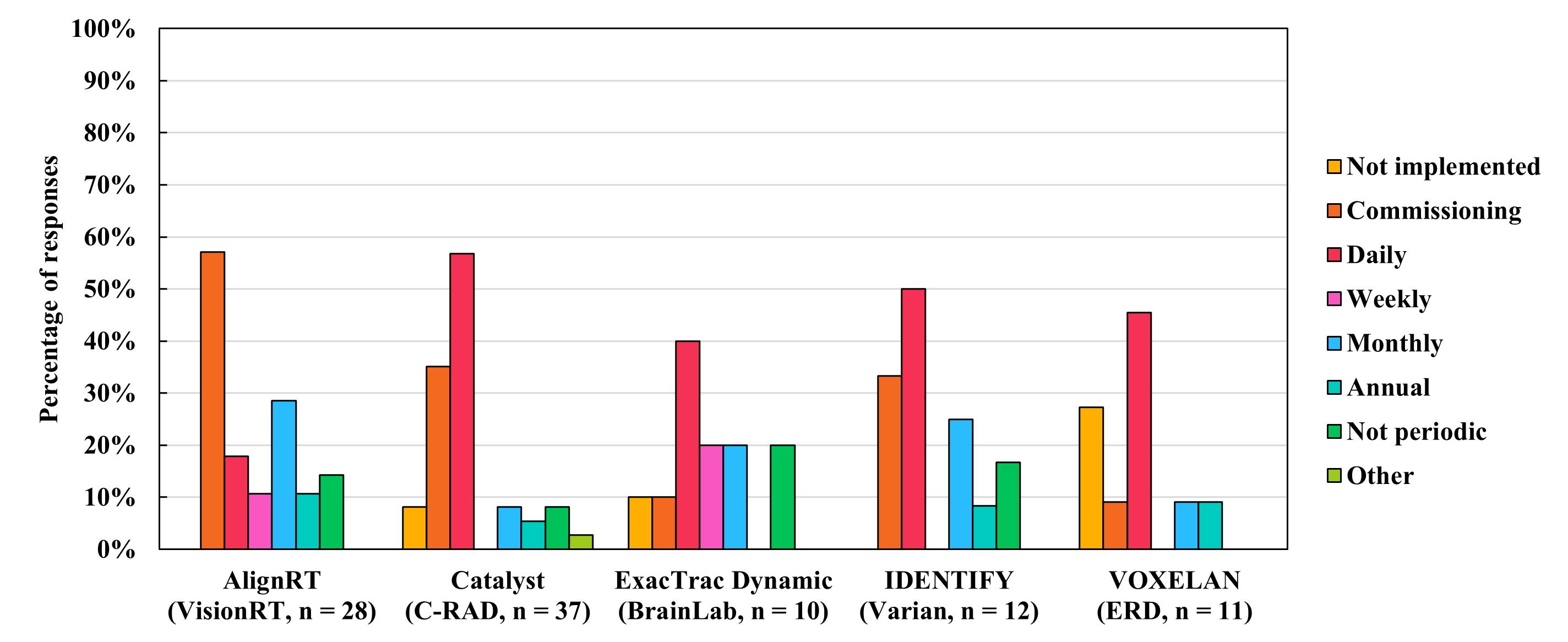


1. Dynamic accuracy


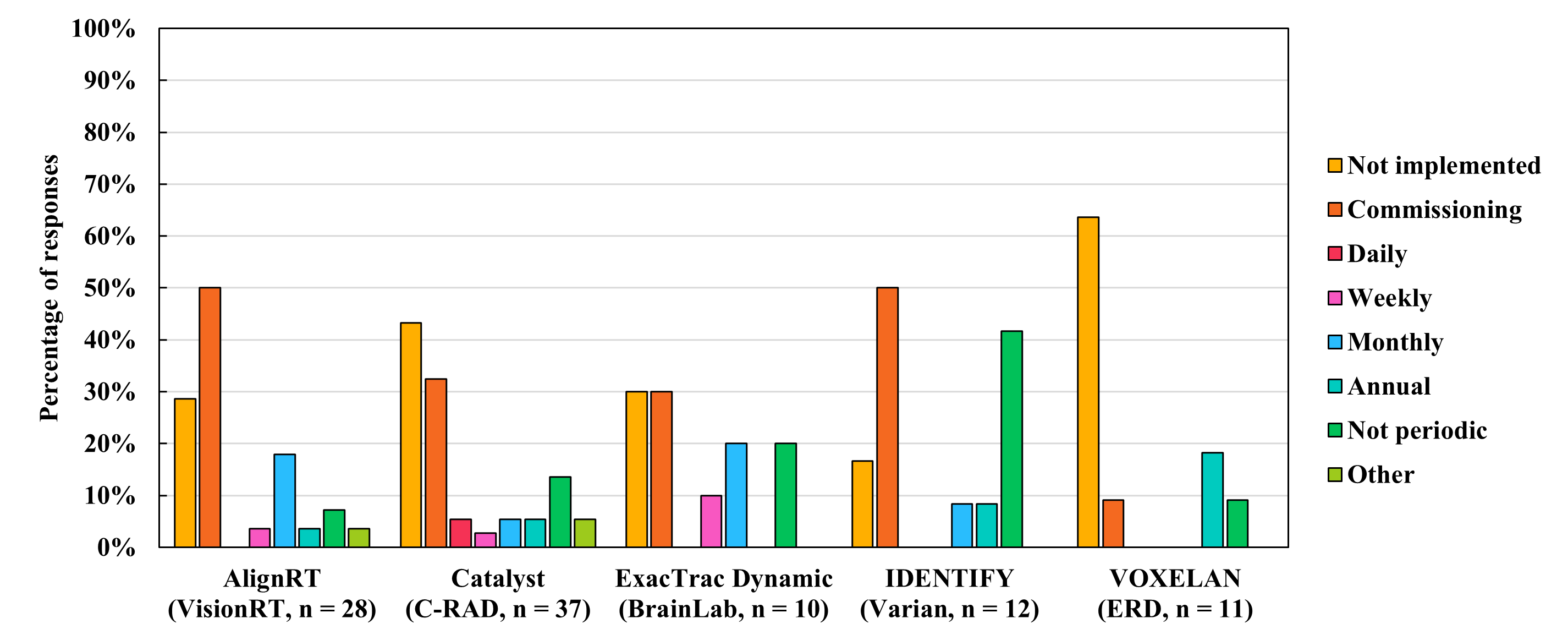


1. Room-light dependence


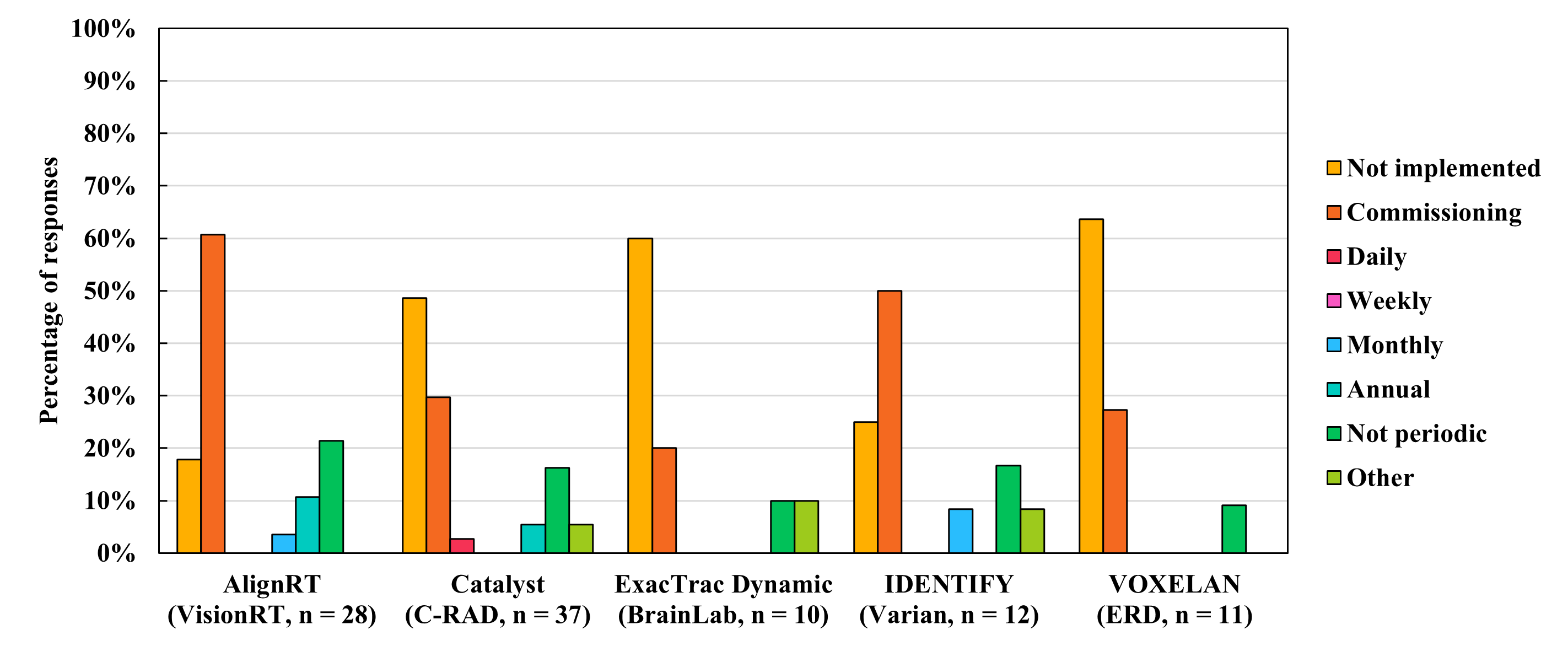


1. Latency


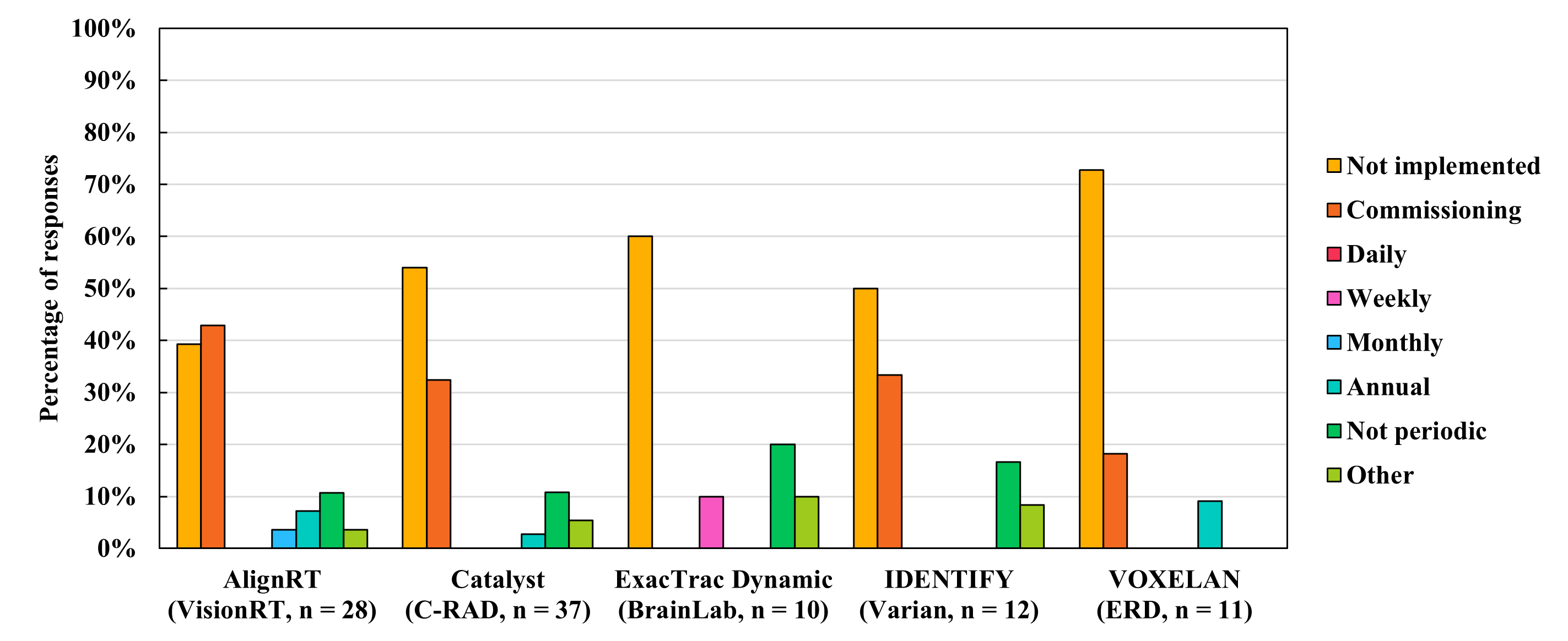


1. End-to-End test


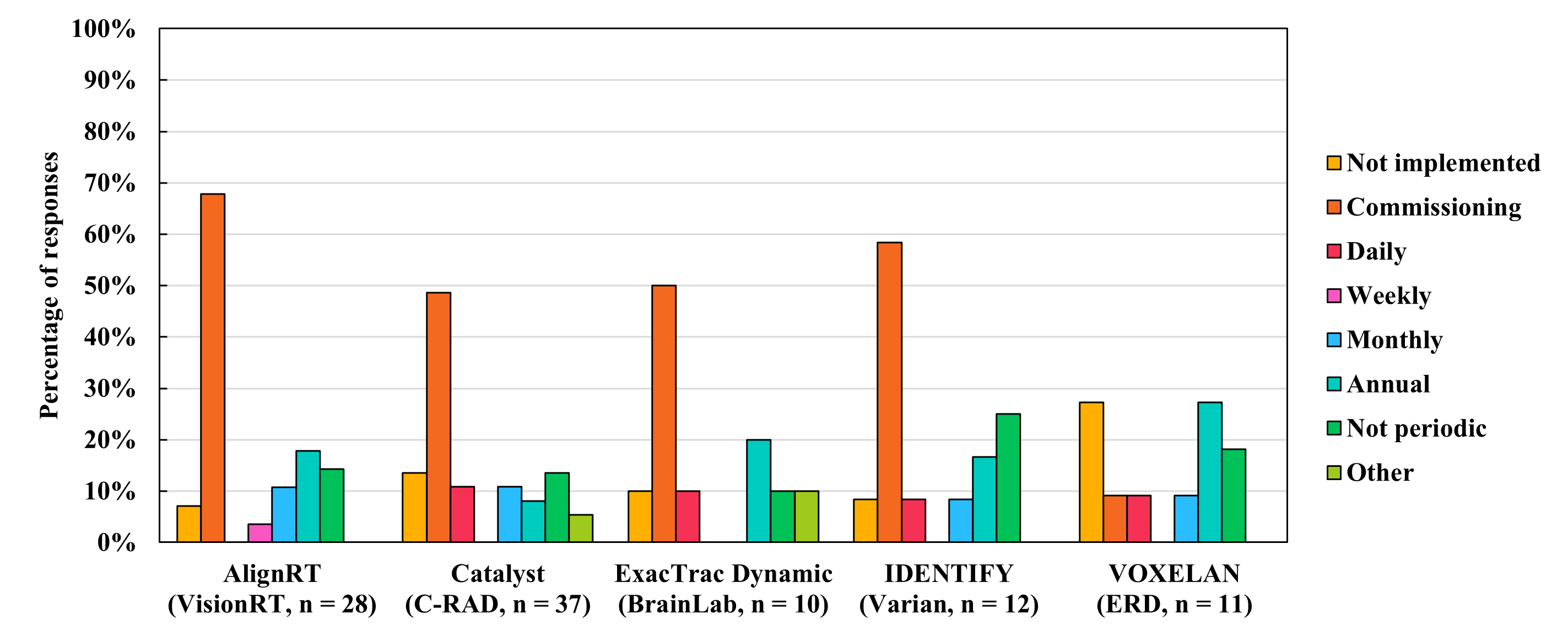


Supplementary figure 5 Frequency distributions of QA test implementation by vendor: (a) Isocenter coincidence, (b) Surface image quality, (c) Static accuracy, (d) Dynamic accuracy, (e) Room light dependence, (f) Latency, and (g) End-to-end test.

1. Commissioning (mechanical)


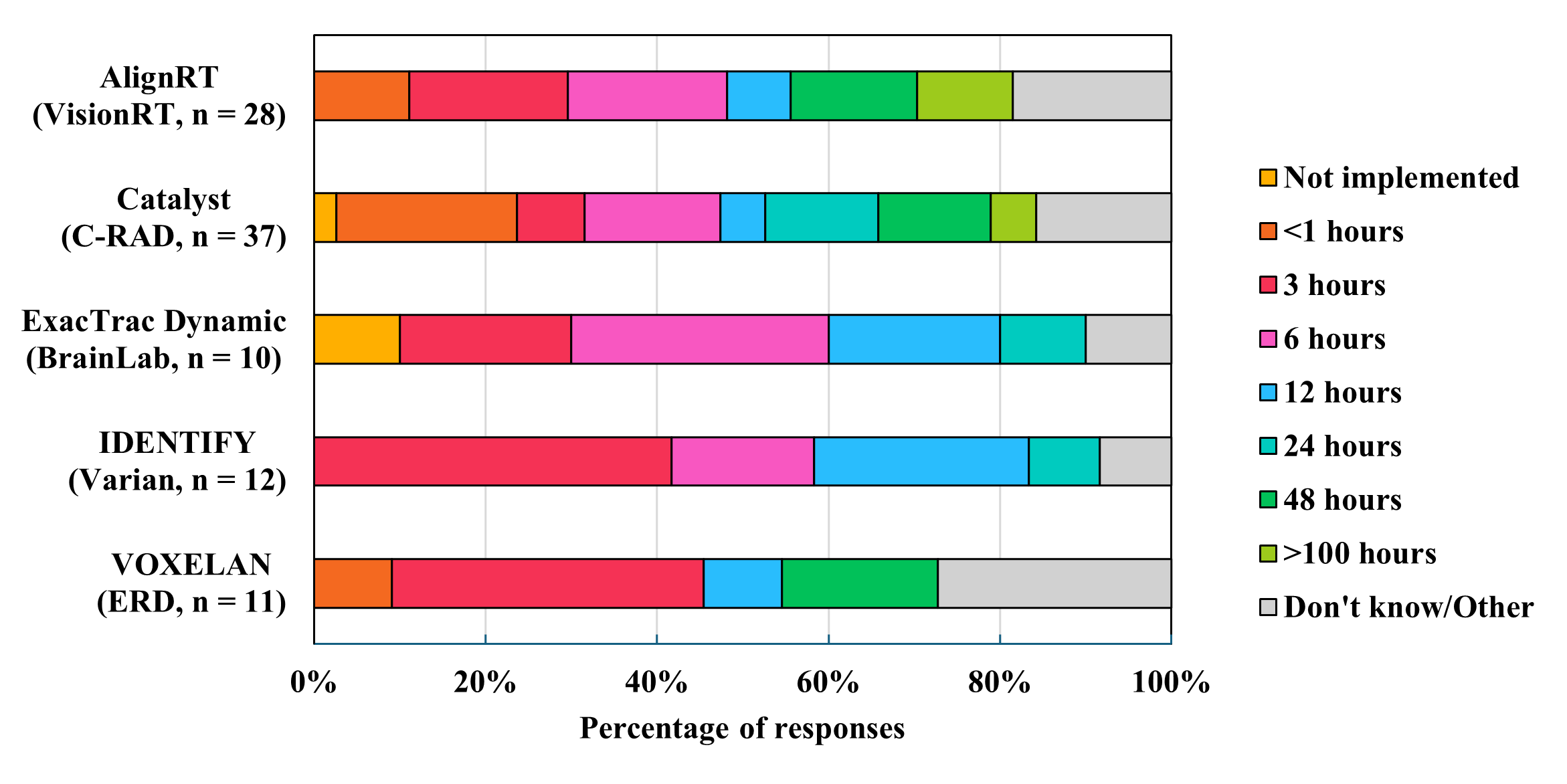


1. Commissioning (clinical)


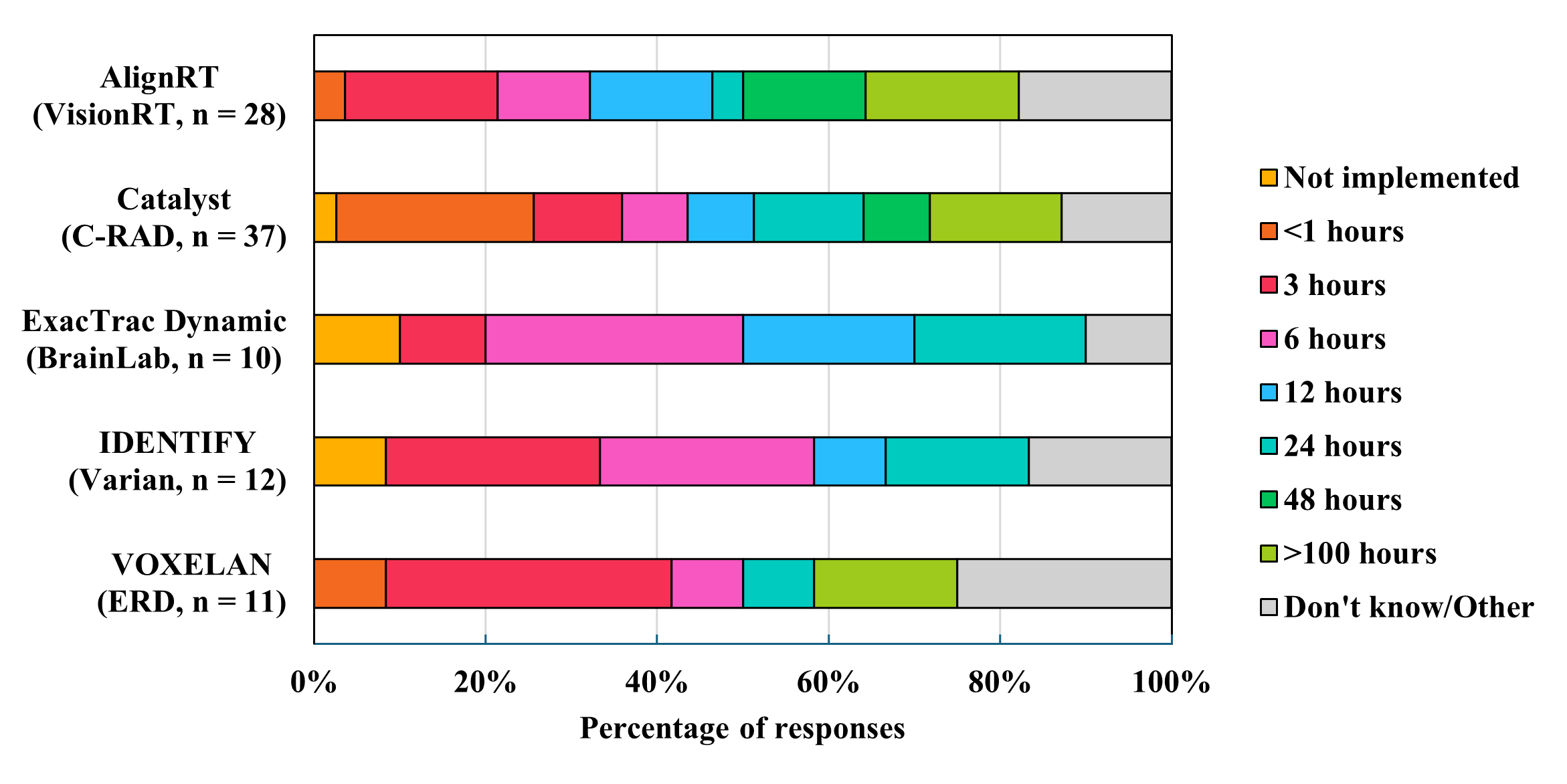


1. Staff training


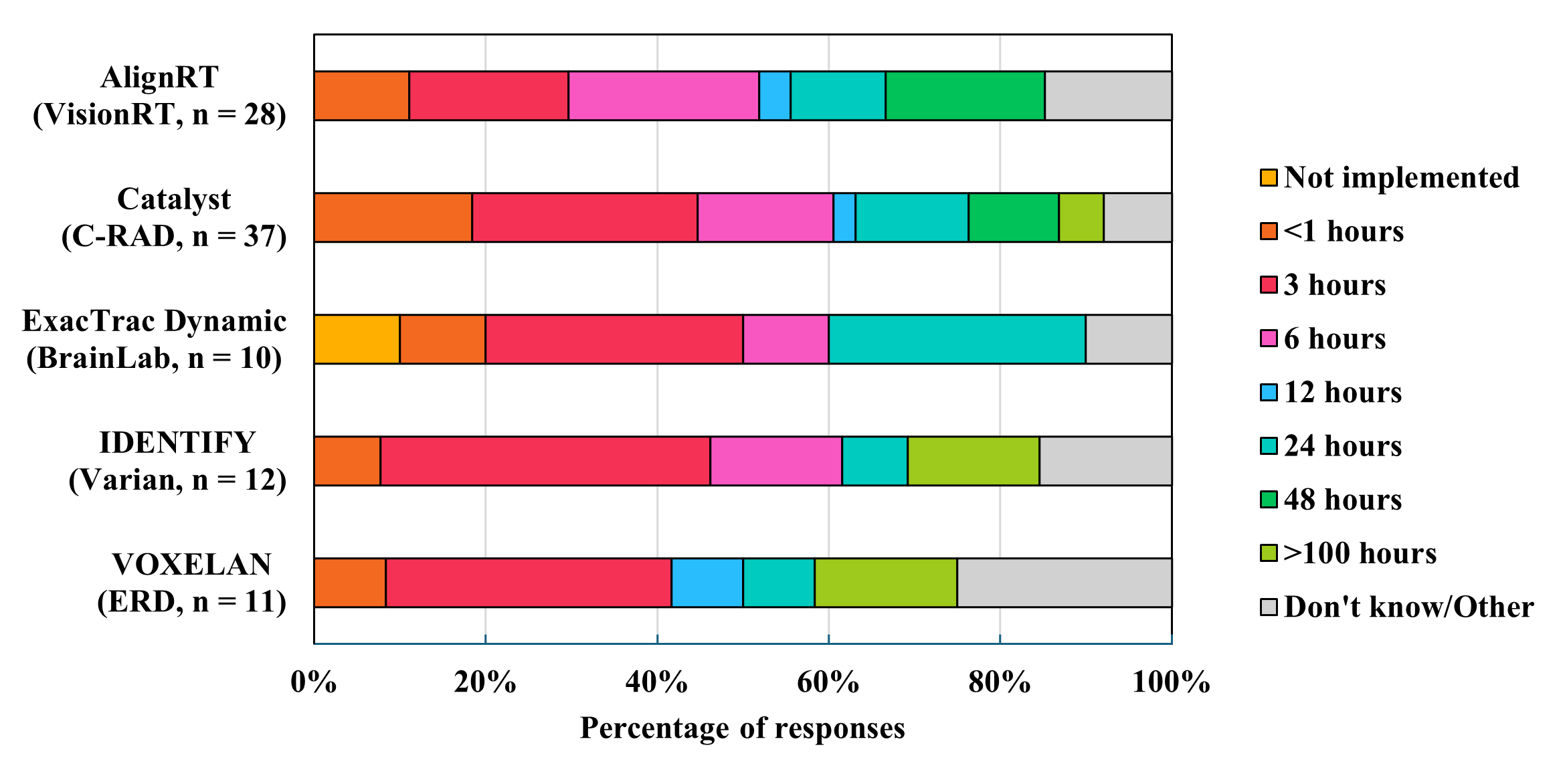


Supplementary figure 6 Frequency distribution of time spent on commissioning, by vendor.

1. Daily-QA


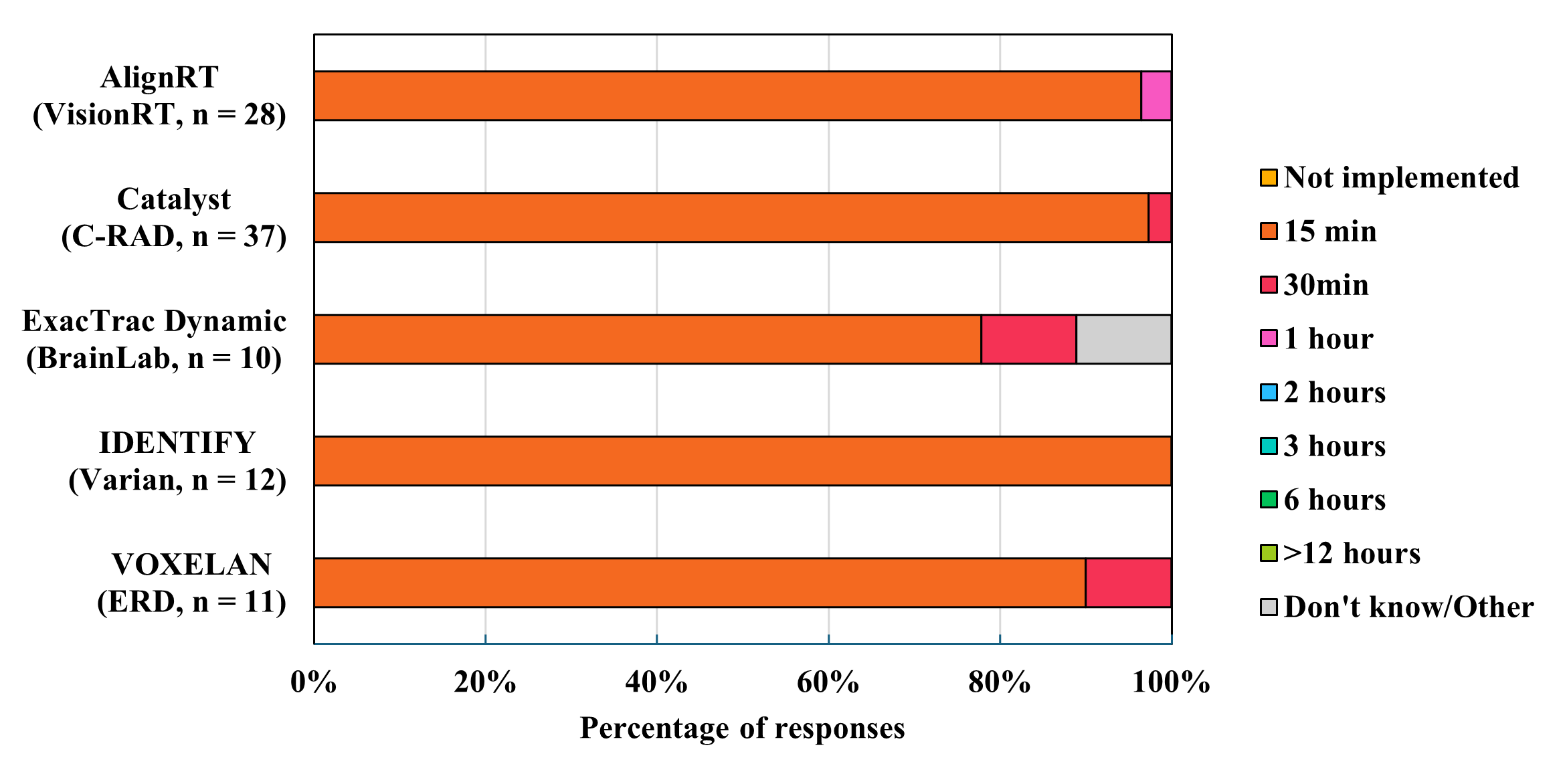


1. Monthly-QA


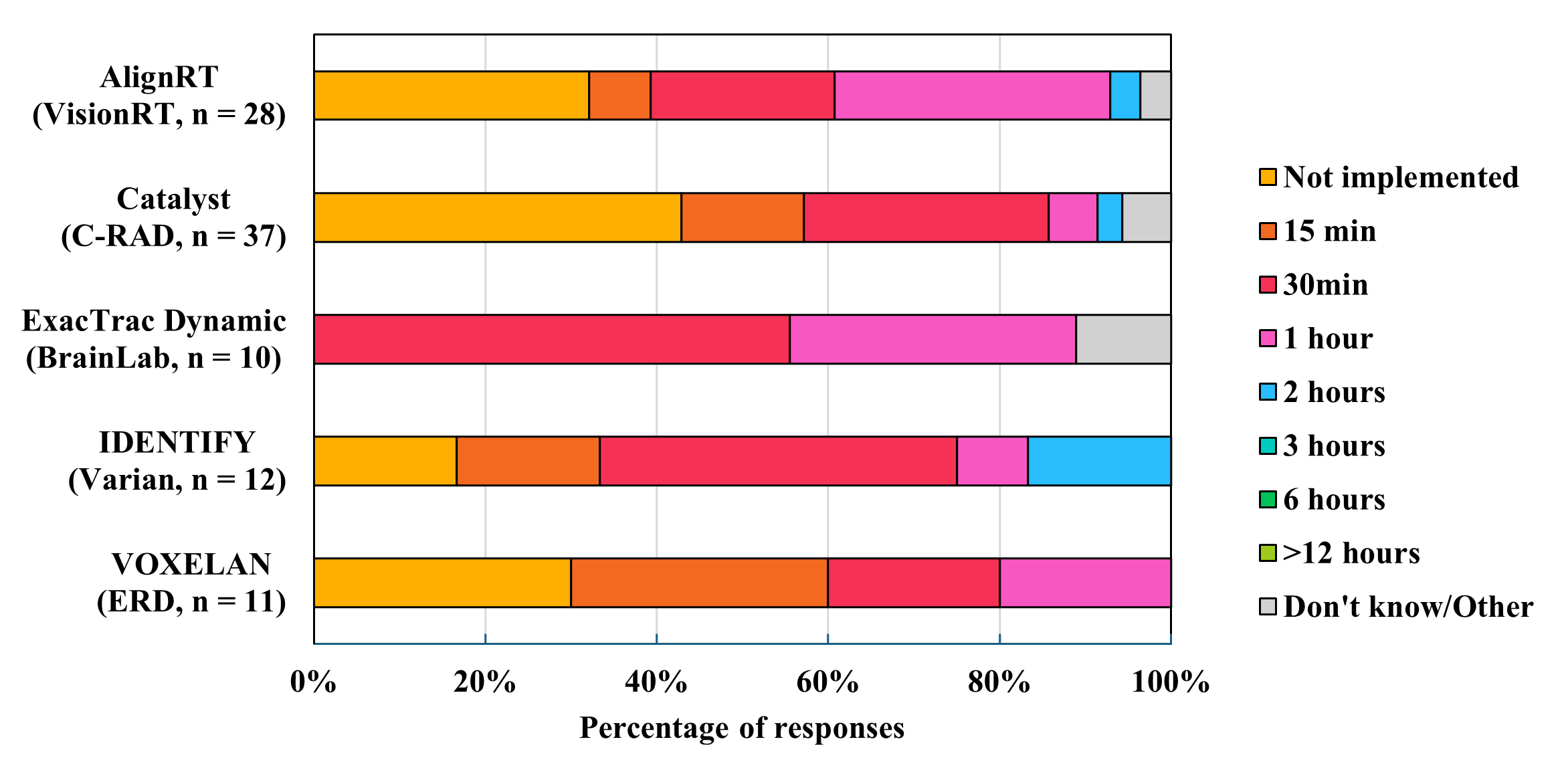


1. Annual-QA


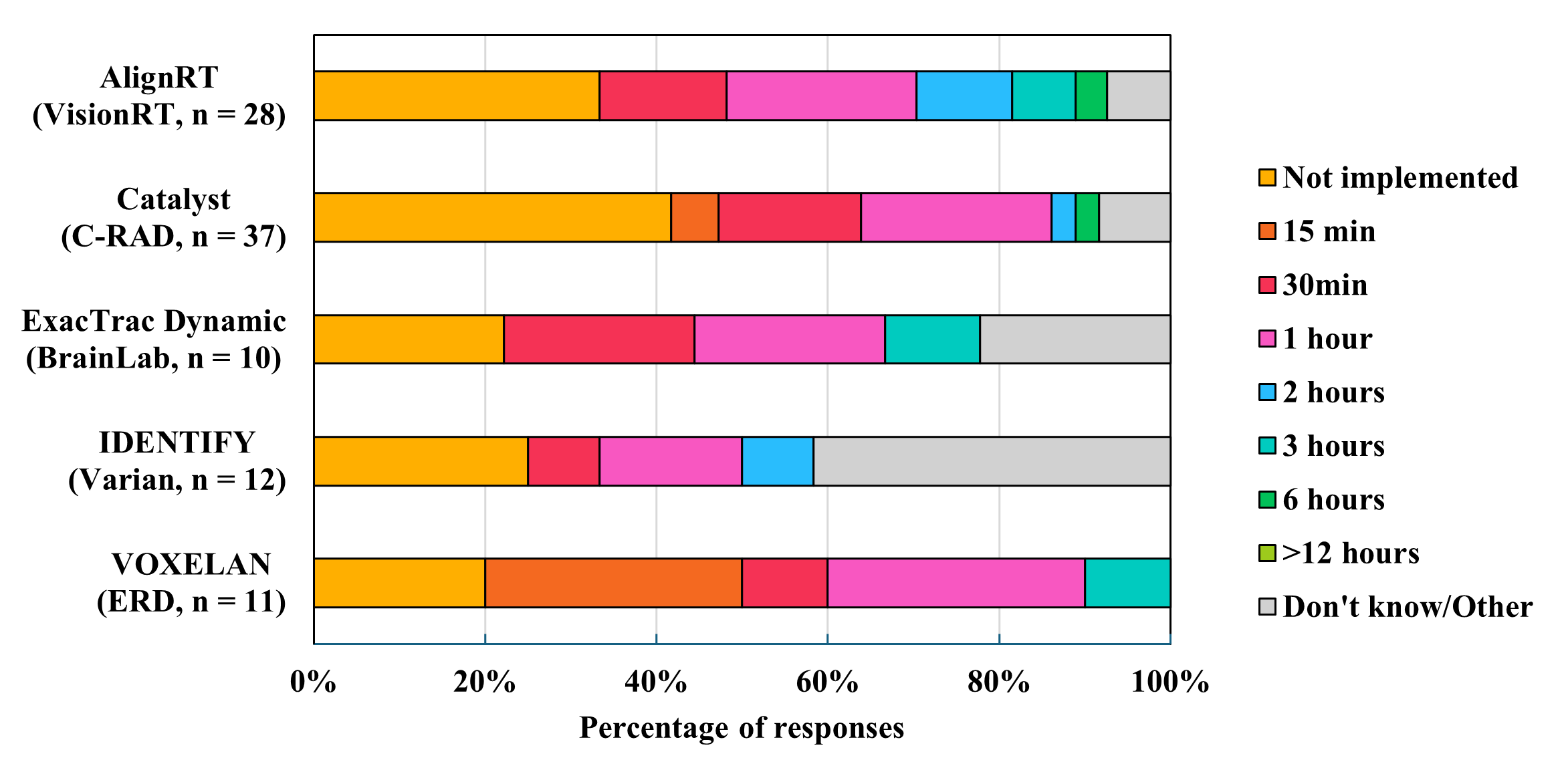


Supplementary figure 7 Frequency distribution of time required for periodic QA tests, by vendor.


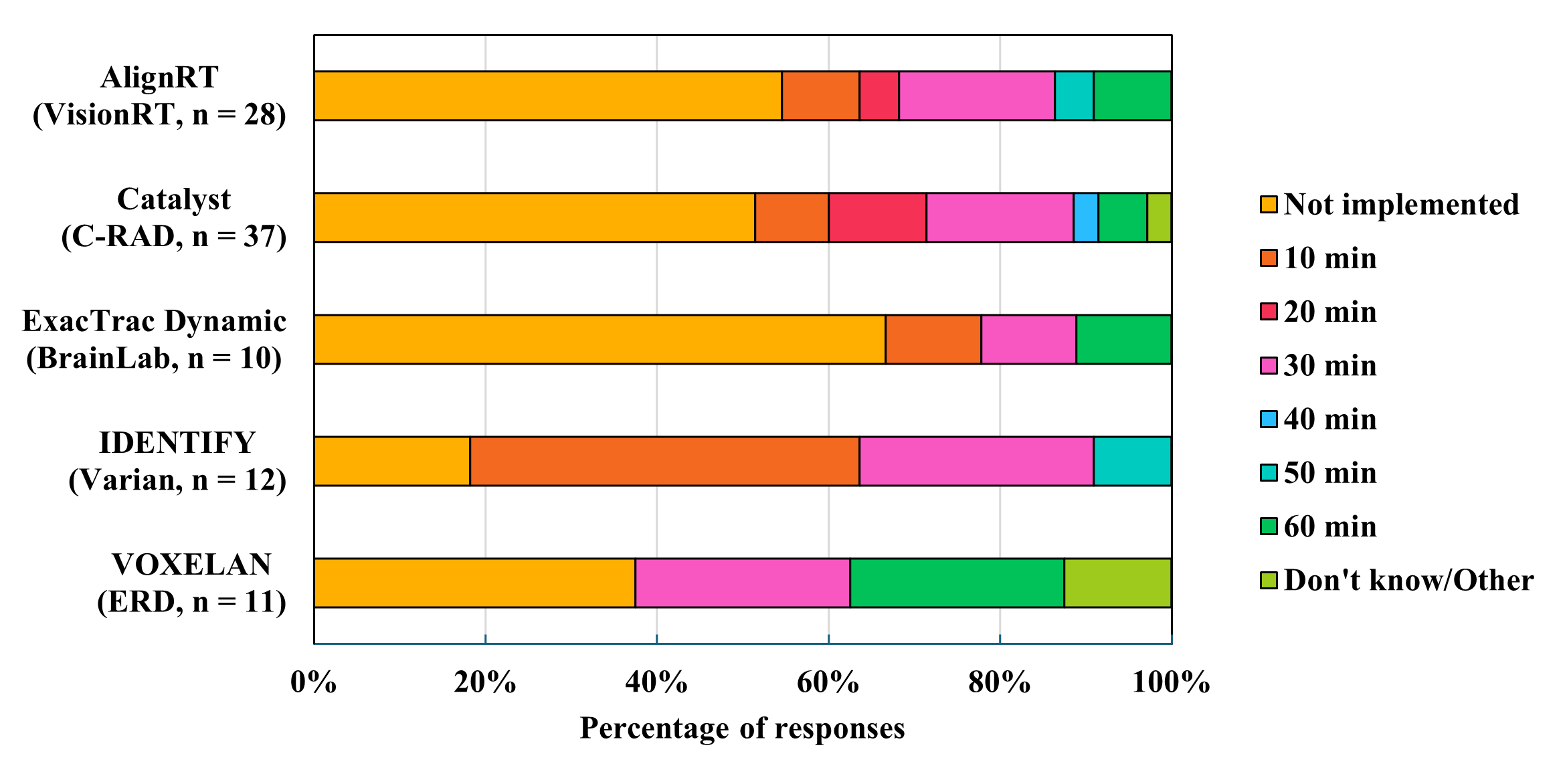


Supplementary figure 8 Warm-up time to ensure stability of the SGRT system, by vendor.


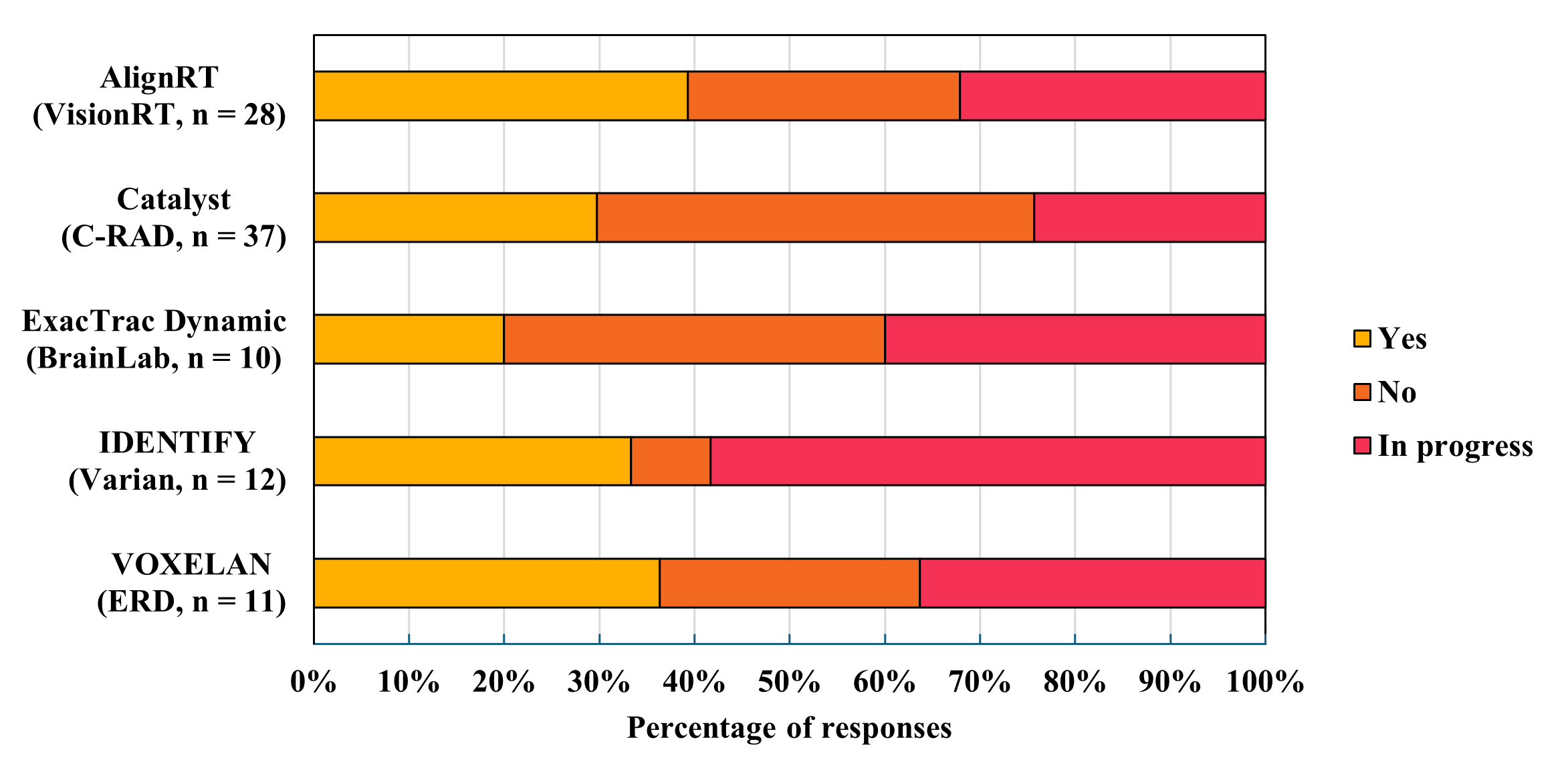


Supplementary figure 9 QA protocols used in clinical practice, by vendor.

1. Reference surface used for patient positioning


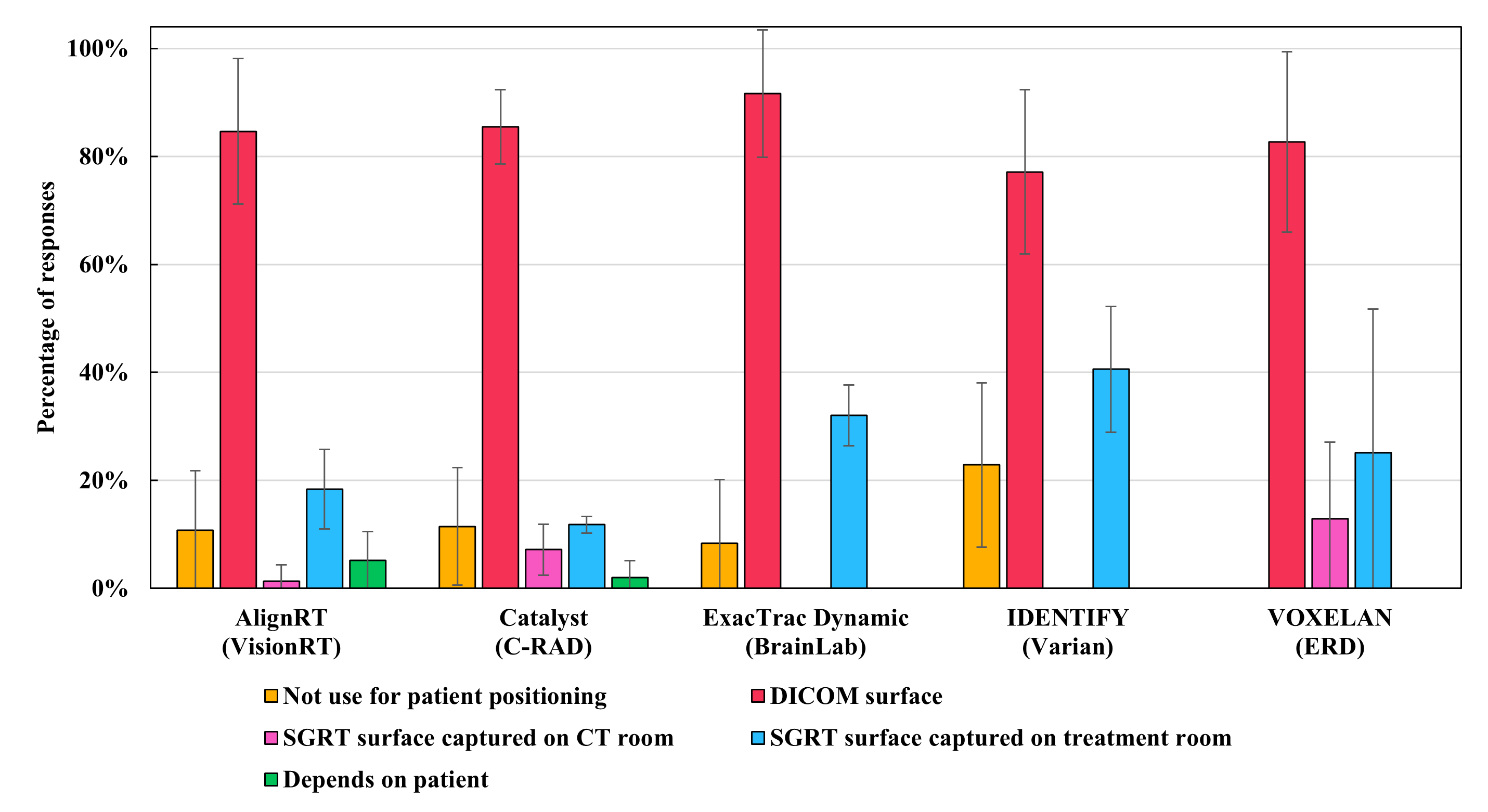


1. Reference surface used for patient monitoring


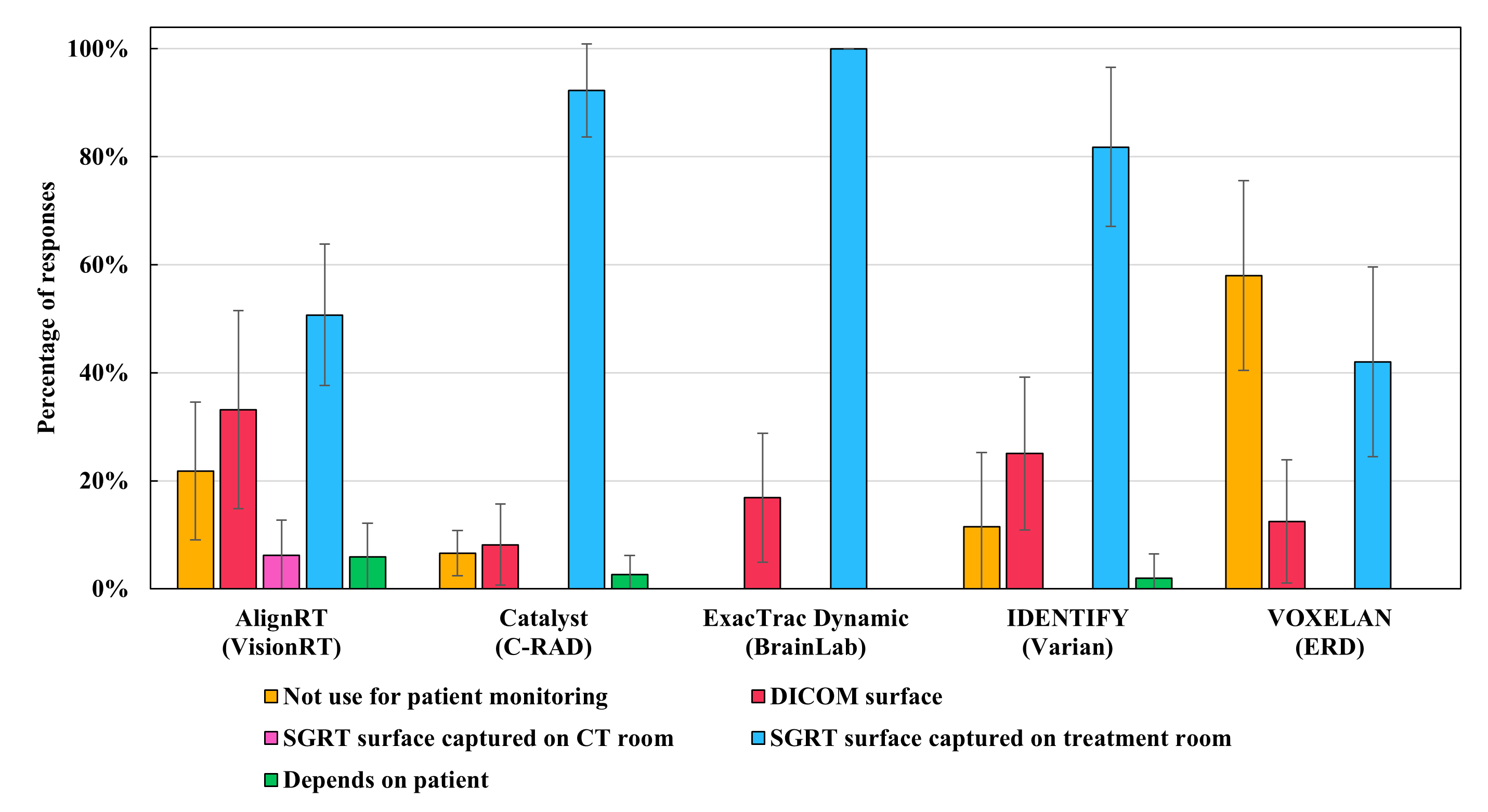


Supplementary figure 10 Reference surfaces used for (a) patient positioning and (b) patient monitoring, aggregated across all treatment sites and summarized by vendor.

1. Breast/chest


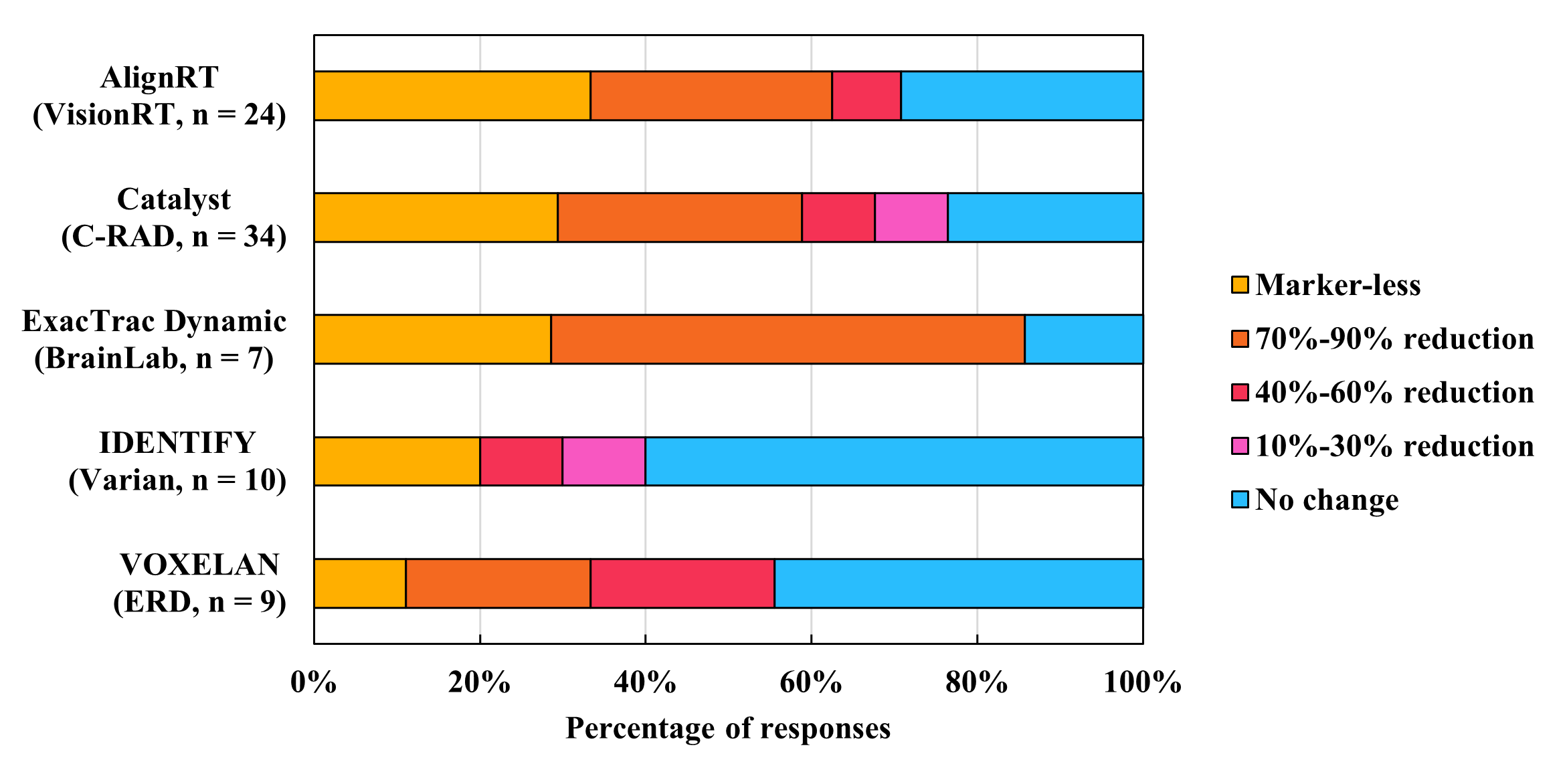


1. Abdomen/Pelvis


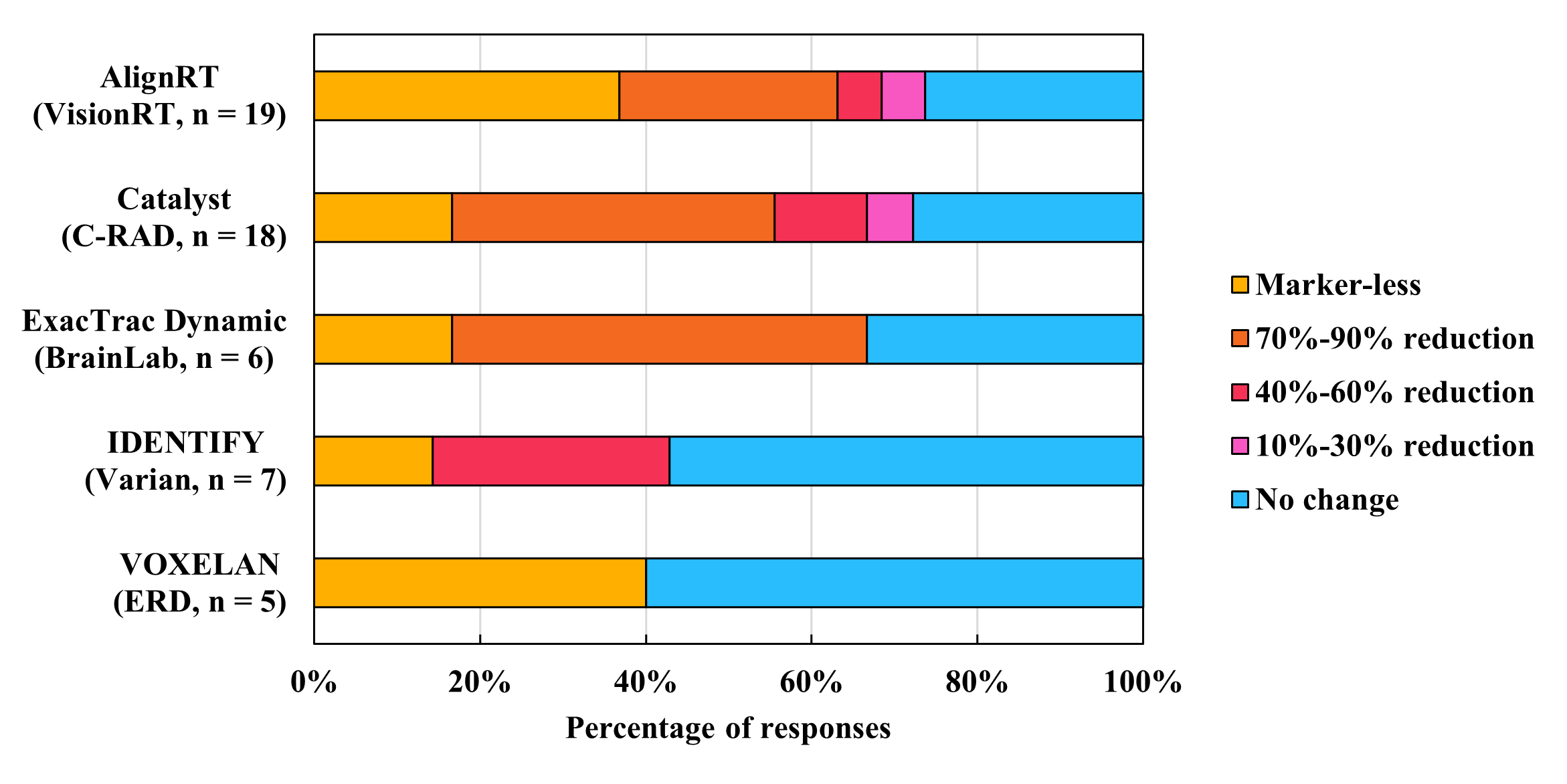


1. Extremity


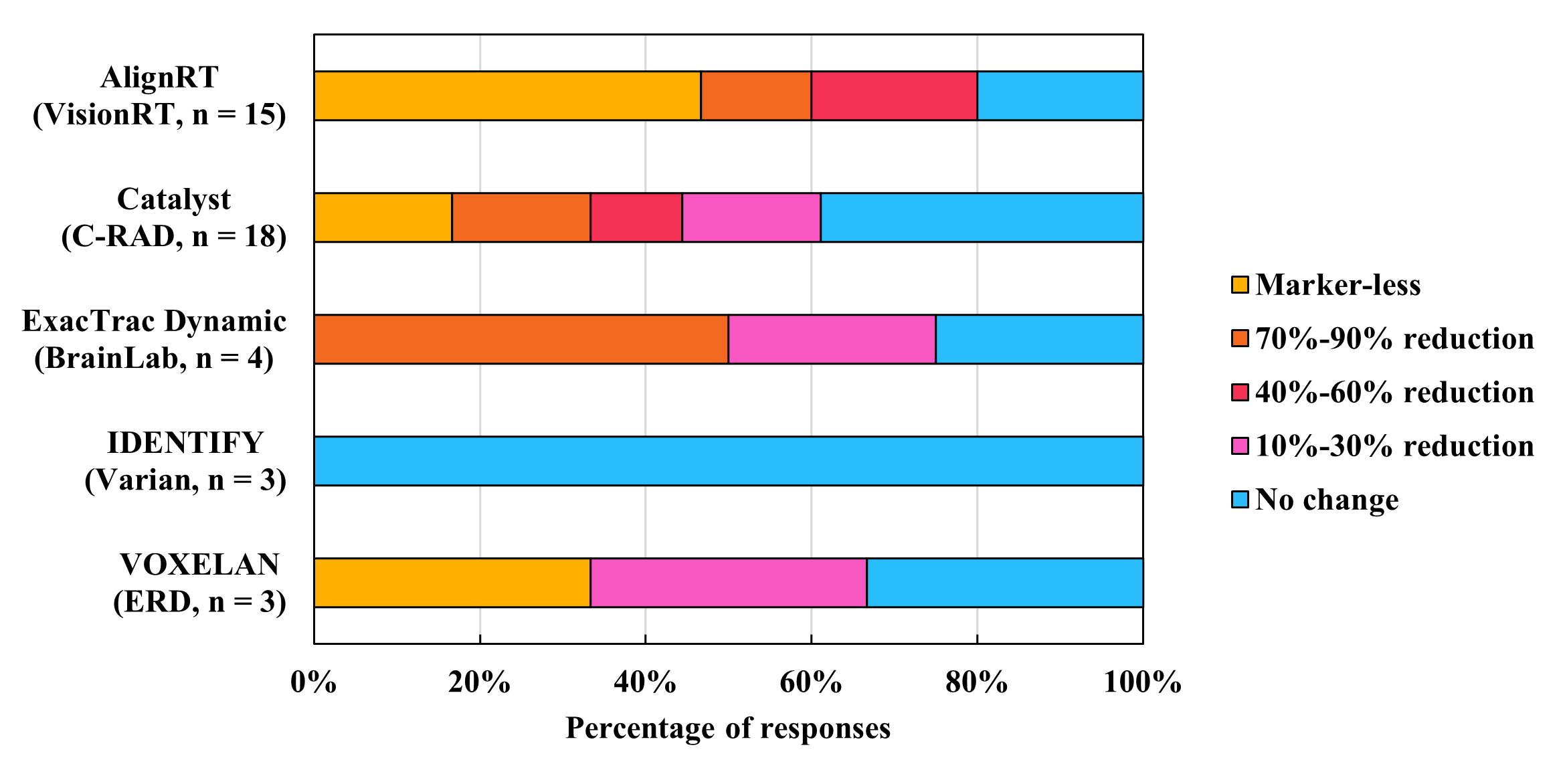


Supplementary figure 11 Changes in skin marking by treatment site after SGRT introduction, categorized by vendor: (a) breast/chest, (b) abdomen/pelvis, and (c) extremity.
